# A New‐Generation Base Editor with an Expanded Editing Window for Microbial Cell Evolution In Vivo Based on CRISPR‒Cas12b Engineering

**DOI:** 10.1002/advs.202309767

**Published:** 2024-04-11

**Authors:** Wenliang Hao, Wenjing Cui, Zhongmei Liu, Feiya Suo, Yaokang Wu, Laichuang Han, Zhemin Zhou

**Affiliations:** ^1^ The Key Laboratory of Industrial Biotechnology (Ministry of Education), School of Biotechnology Jiangnan University 1800 Lihu Avenue Wuxi 214122 China; ^2^ Science Center for Future Foods Jiangnan University Wuxi 214122 China

**Keywords:** base editor, CRISPR‒Cas12b, editing window, protein evolution, synthetic biology

## Abstract

Base editors (BEs) are widely used as revolutionary genome manipulation tools for cell evolution. To screen the targeted individuals, it is often necessary to expand the editing window to ensure highly diverse variant library. However, current BEs suffer from a limited editing window of 5–6 bases, corresponding to only 2–3 amino acids. Here, by engineering the CRISPR‒Cas12b, the study develops dCas12b‐based CRISPRi system, which can efficiently repress gene expression by blocking the initiation and elongation of gene transcription. Further, based on dCas12b, a new‐generation of BEs with an expanded editing window is established, covering the entire protospacer or more. The expanded editing window results from the smaller steric hindrance compared with other Cas proteins. The universality of the new BE is successfully validated in *Bacillus subtilis* and *Escherichia coli*. As a proof of concept, a spectinomycin‐resistant *E. coli* strain (BL21) and a 6.49‐fold increased protein secretion efficiency in *E. coli* JM109 are successfully obtained by using the new BE. The study, by tremendously expanding the editing window of BEs, increased the capacity of the variant library exponentially, greatly increasing the screening efficiency for microbial cell evolution.

## Graphical abstract

This study designed a new‐generation base editor (BE) based on CRISPR/Cas12b. The BE can induce C‐to‐T substitution within an expanded editing window in different microorganisms, providing a powerful targeted mutagenesis tool for microbial synthetic biology.

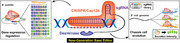

## 1 Introduction

The CRISPR‒Cas system, as an adaptive immune system in bacteria, promotes antiviral defense.^[^ ^1^ ^]^ By reprogramming different CRISPR‒Cas systems (including CRISPR‒Cas9, CRISPR‒Cas12a, and CRISPR‒Cas12f1), precise gene editing (including knock‐out, knock‐in, and point mutation) can be achieved at defined target locations in the genome of a wide variety of cells.^[^ ^2^, ^3^, ^4^ ^]^ These CRISPR‒Cas‐based gene editing methods have facilitated basic biomedical, gene therapy, functional genomic, and synthetic biological researches. Furthermore, the CRISPR interference (CRISPRi) system based on CRISPR‒Cas was developed and has provided an alternative suite of tools for genome regulation.^[^ ^5^ ^]^ In particular, a catalytically inactive Cas9 (dCas9, D10A, and H840A) protein, which lacks endonuclease activity, can be used to flexibly target many loci by using Cas9‐binding guide RNAs. CRISPRi regulation can be used to achieve activation (CRISPRa) or repression by fusing dCas9 with activator or repressor modules.^[^ ^6^, ^7^ ^]^ Similarly, CRISPRi or CRISPRa systems based on CRISPR‒Cas12a have also been developed and successfully used for gene regulation.^[^ ^8^, ^9^ ^]^ In addition to gene regulation, modified CRISPRi systems have been engineered as genome imaging tools for signal amplification in live cells.^[^ ^10^, ^11^, ^12^ ^]^

In the newly developed precise CRISPR‒Cas technology, by using BEs, enables the direct installation of point mutations (C to T or A to G) into genomic DNA by fusing deaminases with Cas nickase (nCas) or dCas.^[^ ^13^, ^14^, ^15^ ^]^ Moreover, new BEs have been developed by fusing different DNA glycosylases (such as uracil‐DNA glycosylase and N‐methylpurine DNA glycosylase).^[^ ^16^, ^17^ ^]^ The discovery of BEs has provided powerful tools for clinical therapeutics, plant breeding, and microbial synthetic biology. However, different application scenarios pose different requirements for the use of BEs in different species. Some human disease‐associated alleles, such as the Alzheimer's disease‐associated gene *APOE4* and the β‐thalassemia locus *HBB*, have multiple Cs around the targeted C within the activity window, and the editing of additional Cs would potentially cause deleterious effects.^[^ ^18^ ^]^ To address these issues, high‐precision BEs are needed for site‐specific single nucleotide replacement. Protein engineering of deaminases has been used to develop BEs with minimal bystander edits.^[^ ^19^, ^20^, ^21^, ^22^, ^23^ ^]^ BEs used in microbial cells are being developed rapidly and widely applied in synthetic biology, including protein engineering,^[^ ^24^, ^25^, ^26^ ^]^ metabolic engineering,^[^ ^27^, ^28^ ^]^ functional gene identification,^[^ ^29^, ^30^ ^]^ and genome mining of novel natural products.^[^ ^31^ ^]^ Unlike in mammalian cells, BEs with expanded editing windows are required for sufficient library construction, through which the efficient bacterial chassis could be obtained through high throughput screening, and the efficient bacterial chassis are the key factor for bio‐manufacturing. The current BEs based on CRISPR‒Cas9 commonly contain an activity window of 5–6 nt, which achieves substitution of up to three amino acids.^[^ ^25^, ^26^, ^28^ ^]^ In addition, BEs often induce synonymous mutations due to codon wobble, which greatly limits the number of amino acid substitutions. Therefore, to a certain extent, expansion of the editing window is a major challenge for BE application in microbial cells. BETTER is one of the base editing tools based on CRISPR‒Cas9 that can generate many genetic combinations of diverse ribosome binding sites (RBSs), 5' untranslated regions, or promoters to regulate the expression of target genes.^[^ ^28^ ^]^ To achieve mutation for eight consecutive Cs (RBS) by BETTER, it is necessary to interlace at least two single‐guide RNAs (sgRNAs) due to its limited editing window.^[^ ^28^ ^]^ Strategies for expanding the editing window for BEs have been proposed through the use of variant Cas proteins,^[^ ^22^, ^32^, ^33^, ^34^ ^]^ screening divergent cytidine deaminases,^[^ ^35^ ^]^ Cas embedding,^[^ ^36^ ^]^ optimization of sgRNA length,^[^ ^25^, ^26^, ^27^ ^]^ and dual deaminase fusion.^[^ ^37^, ^38^ ^]^ However, the editing windows have only been slightly expanded, and the target bases close to the PAM cannot be edited.

Cas12b (also known as C2c1), a third family of class 2 effectors, has been characterized as a dual‐RNA‐guided nuclease containing a single RuvC domain.^[^ ^39^, ^40^, ^41^ ^]^ At present, Cas12b derived from *Alicyclobacillus acidoterrestris* (AacCas12b) is most well studied.^[^ ^39^, ^40^ ^]^ Similar to Cas12a, Cas12b recognizes a distal 5'‐T‐rich PAM sequence, in contrast to the proximal 3'‐G‐rich PAM favored by Cas9. However, the cleavage activity of Cas12b requires both crRNA and tracrRNA, a feature that is in sharp contrast to Cas12a, which only requires crRNA. Similar to Cas9, an engineered sgRNA generated by covalently fusing crRNA and tracrRNA can also guide Cas12b to cleave target DNA in a staggered seven‐nucleotide break.^[^ ^40^ ^]^ Generally, Cas12b proteins, such as Cas12b (1108 aa) from *Bacillus hisashii*, Cas12b (1129 aa) from *Alicyclobacillus acidoterrestris*, and Cas12b (1129 aa) from *Alicyclobacillus acidiphilus*, are smaller than the most widely‐used Cas9 (1369 aa) from *Streptococcus pyogenes*, Cas12a (1353 aa) from *Acidaminococcus sp*, and Cas12a from *Lachnospiraceae bacterium*, and have minimal off‐target effects.^[^ ^39^ ^]^ The CRISPR‒Cas12b system has been used for genome editing and gene regulation in mammalian and plant cells.^[^ ^42^, ^43^, ^44^ ^]^ However, there are few reports on the potential applications of CRISPR‒Cas12b‐based genome (base) editing and gene regulation in industrial microorganisms. We speculate that the smaller size of the Cas12b protein may alleviate the steric hindrance of deaminase, thereby expanding the editing window of BEs. Moreover, the development of BEs based on Cas12b would expand the collection of non‐Cas9‐derived BEs.^[^ ^45^ ^]^

Here, we described a new CRISPR‒Cas12b‐based BE with an expanded editing window, covering the entire protospacer or more. BhCas12b was selected because it exhibited high editing activity.^[^ ^43^ ^]^ Three key amino acids (D574, E828, and D952) affecting the nuclease activity of BhCas12b were identified through primary sequence alignment and molecular docking of BhCas12b and sgRNA, and a catalytically inactive BhCas12b (dBhCas12b) was obtained. The efficiency of the dBhCas12b‐based CRISPRi system with broad‐spectrum repression was confirmed, and dBhCas12b‐based BEs with an expanded editing window (≈19 nt) were constructed in *B. subtilis*. The dBhCas12b‐based BE also worked well in *E. coli* BL21 (DE3) and *E. coli* JM109. Surprisingly, the BE exhibited an astonishing editing window (≈43 nt), covering the protospacer and more, and the mutant library would increase exponentially compared to the common BEs. A spectinomycin‐resistant *E. coli* strain BL21 (DE3) and a 6.49‐fold increased protein secretion efficiency of *E. coli* JM109 were efficiently obtained by using the new BE. Our study tremendously expanded the editing window of BEs, providing a brand‐new genomic mutagenesis tool for accelerating cell evolution.

## 2 Results

### 2.1 Design of CRISPR‒Cas12b for Genome Editing in *B. Subtilis*

**Figure 1:**
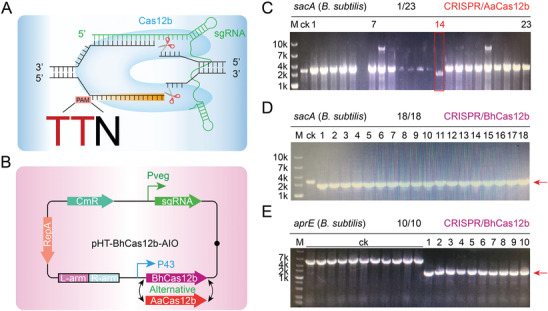
CRISPR‐BhCas12b‐based gene deletion in *B. subtilis*. A) A schematic illustration of CRISPR‒Cas12b. B) Schematic of the CRISPR‐AaCas12b and CRISPR‐BhCas12b systems. C) CRISPR‐AaCas12b system‐mediated deletion of *sacA*. The deletion efficiency was 1/23. The positive mutant is framed in a red rectangle. D) CRISPR‐BhCas12b system‐mediated deletion of *sacA*. The deletion efficiency was 18/18. E) CRISPR‐BhCas12b system‐mediated deletion of *aprE*. The deletion efficiency was 10/10. The lane “ck” shows the PCR product from the wild‐type strain. The relevant sgRNA sequences are listed in Table S4 (Supporting Information).

The CRISPR‒Cas12b system derived from AaCas12b and BhCas12b has been well characterized in human cells, both functioning at 37 °C,^[^ ^42^, ^43^ ^]^ and Cas12b was reported to generate staggered seven‐nucleotide breaks distal to the 5'‐T‐rich PAM sequence (**Figure** **1**A). To construct CRISPR‒Cas12b‐assisted genome editing systems in *B. subtilis*, two codon‐optimized Cas12b sequences (AaCas12b and BhCas12b) and their cognate chimeric single‐guide RNAs (sgRNAs) were synthesized. Two all‐in‐one (AIO) plasmids harboring AaCas12b, BhCas12b and the corresponding sgRNA were constructed, where Cas12b and sgRNA were regulated by the strong constitutive promoters P43 and P*_veg_*, respectively (Figure 1B). The plasmids were transformed into *B. subtilis* for gene *sacA* deletion. As shown in Figure 1C,D, AaCas12b exhibited low deletion efficiency, while BhCas12b generated 100% deletion efficiency, suggesting that BhCas12b was more robust than AaCas12b for genome editing in *B. subtilis*. To further determine the robustness of CRISPR‒BhCas12b, another gene, *aprE*, was selected as the target for deletion. As a result, the deletion efficiency at the *aprE* locus was also 100% (Figure 1E), demonstrating that CRISPR‒BhCas12b is a potential tool for bacterial genome editing.

### 2.2 Catalytically Inactive Cas12b Isolation

**Figure 2:**
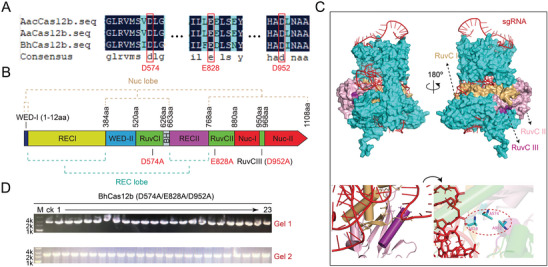
Identification of key catalytic residues for BhCas12b. A) Sequence homology of AacCas12b, AaCas12b, and BhCas12b. B) Schematic illustration of the BhCas12b domain structure, including the positions of the mutant catalytic residues. C) Surface representation of the BhCas12b‐sgRNA complex. Residues A574, A828, and A952 form a DNA catalytic pocket. RuvC I, RuvC II, and RuvC III are shown in tan, pink, and purple, respectively. D) Identification of the *sacA* deletion efficiency of the BhCas12b variant (D574A/E828A/D952A). The lane “ck” shows the PCR product from the wild‐type strain.

BEs commonly involve the partnership of a catalytically impaired Cas protein (such as dCas or nCas) with a DNA deaminase. To construct deactivated BhCas12b, potential catalytic residues were identified based on the sequence homology of AacCas12b (PDB: 5WQE),^[^ ^39^ ^]^ AaCas12b,^[^ ^42^ ^]^ and BhCas12b,^[^ ^43^ ^]^ and three residues (D574, E828, and D952) on BhCas12b were selected as mutant candidates based on deactivated AacCas12b (**Figure** **2**A). The positions of the three catalytic residues were also analyzed by mapping the domain organization (Figure 2B). Molecular docking of BhCas12b and sgRNA indicated that the three residues located in nuclease activity domains, RuvC I (yellow), RuvC II (pink), and RuvC III (purple), were far away from the sgRNA (Figure 2C). Therefore, we predicted that mutation of the three residues would not affect sgRNA binding. The three residues were substituted by alanine progressively, and a highly efficient CRISPR‐BhCas12b‐based probe plasmid (the AIO plasmid described above for the deletion of the *sacA* locus) was used to identify whether the mutations would affect the deletion activity of CRISPR‒BhCas12b. As shown in Figure S1A,B (Supporting Information), BhCas12b (D574A) and BhCas12b (D574A/E828A) lost most of the deletion efficiency, while BhCas12b (D952A) still retained 28.57% editing efficiency for *sacA* deletion (Figure S1C, Supporting Information), suggesting that it may need to be combined with two other point mutations (D574A and E828A) to fully repress its DSBs effect. Through testing, we found that BhCas12b (D574A/E828A/D952A) (hereafter referred to as dBhCas12b) lost the deletion efficiency completely (Figure 2D).

### 2.3 Engineering of CRISPR‒BhCas12b for Efficient Repression of Gene Transcription

**Figure 3:**
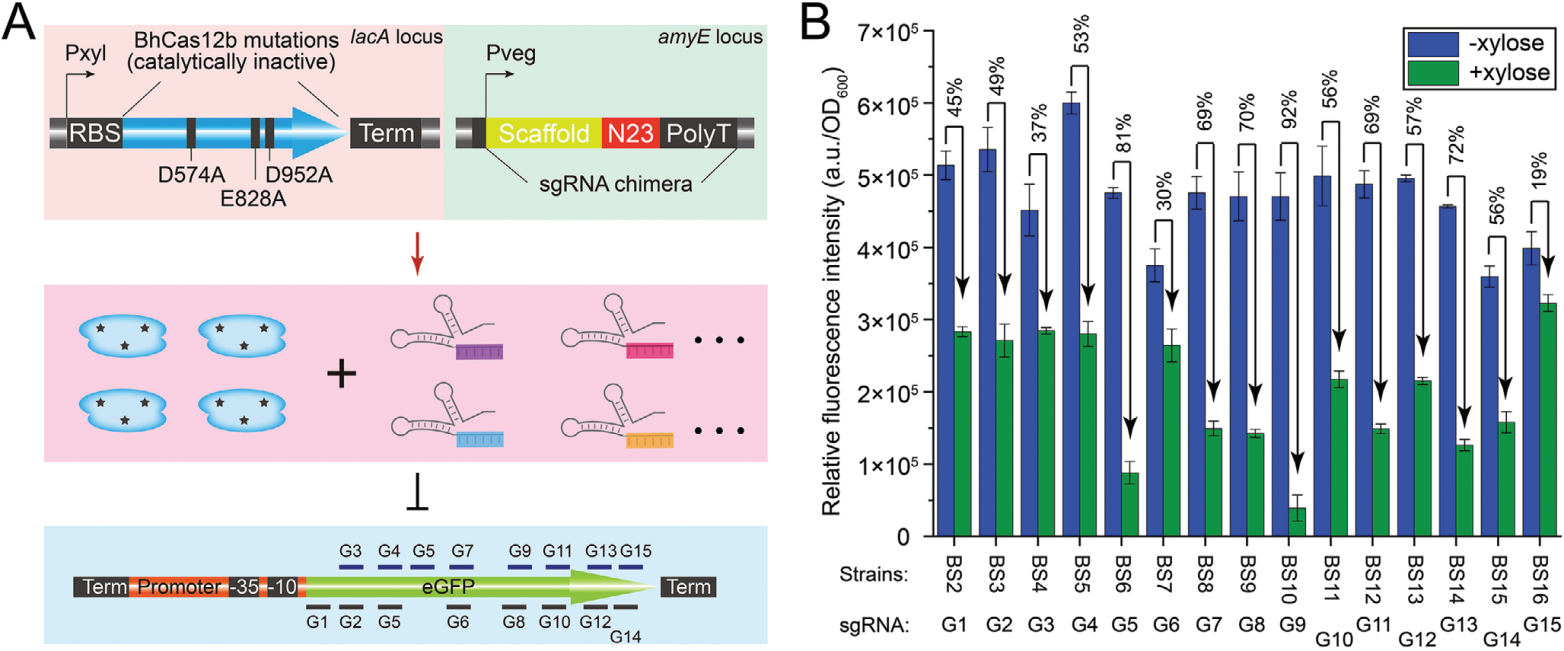
Construction of the dBhCas12b‐based CRISPRi system for repressing gene expression in *B. subtilis*. A) Schematic of the two cassettes used to implement CRISPRi in *B. subtilis*. The expression of dBhCas12b was regulated through a xylose‐induced promoter, and the sgRNA was expressed through the strong constitutive promoter P*_veg_*. B) RFI of the recombinant strains with or without 1% inducer xylose. Numbers above each bar indicate the fluorescence repression rate relative to the control strains that were not induced by xylose. Values and error bars reflect the mean ± s.d. of three independent biological replicates (n = 3). The relevant sgRNA sequences are listed in Table S4 (Supporting Information).

To verify whether the mutations in dBhCas12b affect sgRNA binding and DNA recognition, gene transcription repression using the CRISPR‒dBhCas12b system was performed. The integration vector pAX01 with the strong inducible promoter P*_xylA_* was used for dBhCas12b expression and then integrated into the *lacA* locus of *B. subtilis*, resulting in the recombinant strain BS1 (**Figure** **3**A). Fifteen sgRNAs targeting different positions of the enhanced green fluorescent protein (eGFP) coding region were designed and expressed using the integration vector pDGT with the promoter P*_veg_* (Figure S2, Supporting Information). The fifteen sgRNAs (G1 to G15) were integrated into the *amyE* locus of BS1, generating fifteen recombinant strains (BS2 to BS16, Figure 3B). A fluorescence‐based reporter plasmid, pB‐P43‐eGFP, was transformed into the recombinant strains, and the cell density (OD_600_) and fluorescence intensity (FI) were determined. As shown in Figure S3A (Supporting Information), all the recombinant strains exhibited higher cell density with 1% xylose than those without xylose because sufficient xylose served as both an inducer and a supplementary carbon source. The total FI of most recombinant strains with 1% xylose was lower, indicating that the expressed dBhCas12b guided by sgRNAs strongly repressed the transcription of the eGFP gene (Figure S3B, Supporting Information). The relative fluorescence intensity (RFI) was calculated, and a broad‐spectrum repression rate from 19% to 92% was observed (Figure 3B). These results demonstrated that the mutations in dBhCas12b did not affect sgRNA binding and DNA recognition and that dBhCas12b was successfully repurposed for transcriptional repression, resulting in a dBhCas12b‐based CRISPRi system. Moreover, based on the analysis of repression effect of six promoters (Table S4, Supporting Information) from *B. subtilis*, we found that the system can also efficiently repress the transcription initiation of gene (Figure S4, Supporting Information).

### 2.4 Construction of dBhCas12b‐Based BEs with Expanded Editing Windows

**Figure 4:**
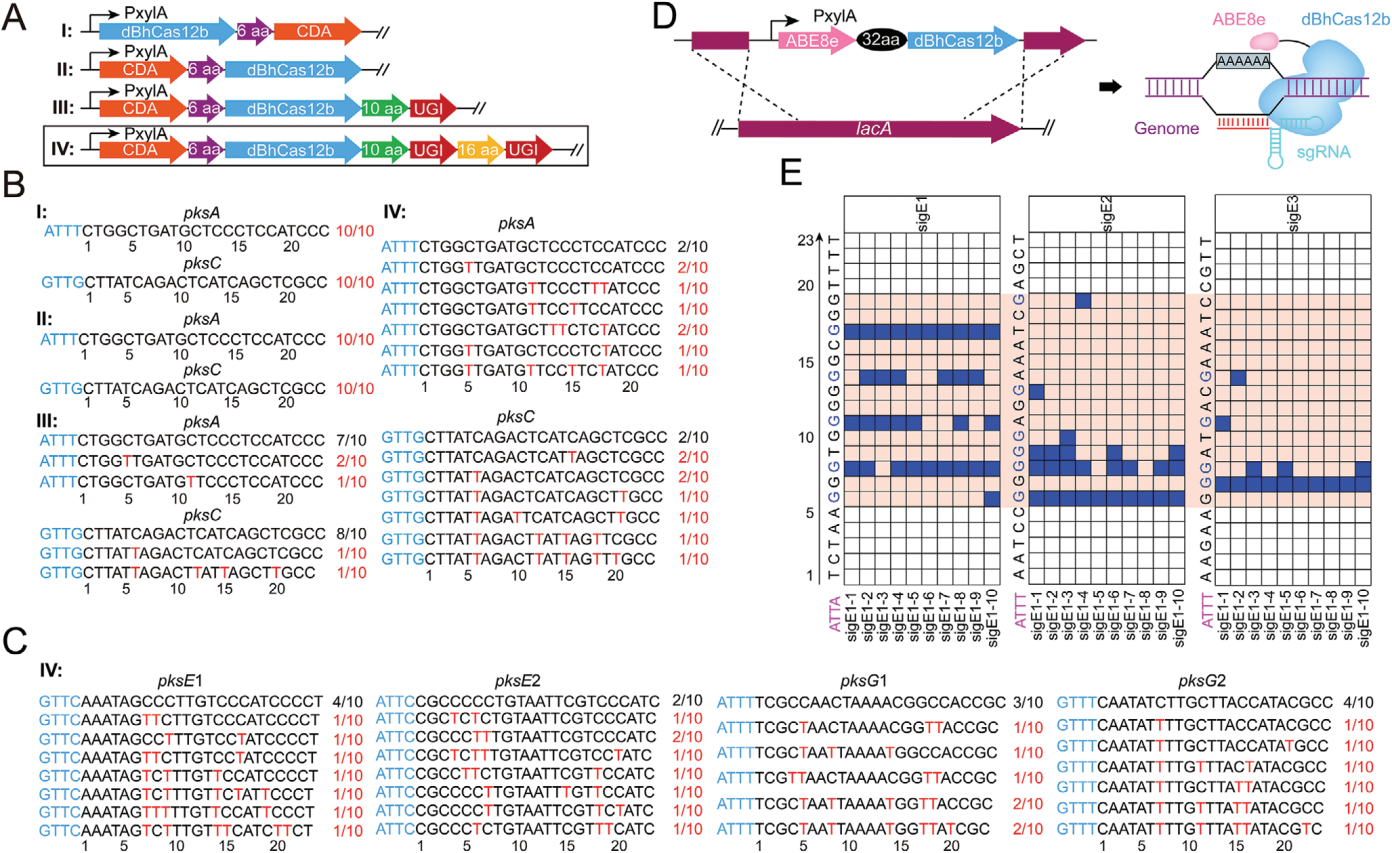
Design and construction of dBhCas12b‐based CBEs in *B. subtilis*. A) Architectures of different CBEs. The proteins were fused using linkers with different lengths. aa, amino acid. B) Investigation of the editing performance of the CBEs for *pksA* and *pksC*. C) Editing performance of CBE‐IV for *pksE* and *pksG*. D) Architecture of the dBhCas12b‐based ABE. The P*_xylA_*‐ABE8e‐dBhCas12b expression cassette was inserted into the *lacA* locus of *B. subtilis*. E) The editing efficiency of the ABE targeting different sites of *sigE* (sigE1, sigE2, and sigE3). Partial bases mutated to Cs by the CBE are shown in red Ts; partial bases mutated to As by the ABE are shown in bright blue Gs; PAMs are highlighted in pink; the editing window is indicated in orange.

The dBhCas12b was used to construct CBEs, and four types of CBEs were designed and constructed. The cytidine deaminase from *Petromyzon marinus* (PmCDA) was fused to the N‐ or C‐terminus of dBhCas12b, producing CBE‐I and CBE‐II (**Figure** **4**A). To improve the conversion efficiency, CBE‐II was further fused to one or two copies of uracil DNA glycosylase inhibitor (UGI, inhibiting the reverse mutation of U to C), resulting in CBE‐III and CBE‐IV (Figure 4A). The four CBEs were cloned and inserted into the integration vector pAX01 and then integrated into the *lacA* locus of *B. subtilis*, resulting in four recombinant strains (BS38 to BS41). Two sgRNAs targeting *pksA* and *pksC* were designed and cloned and inserted into the pHYT vector, where the transcription of sgRNAs is regulated by the strong constitutive promoter P*_veg_*. Each sgRNA was transformed into the recombinant strains BS38, BS39, BS40, and BS41. To verify the reliability of the four CBE systems for base editing, the editing efficiency was verified at the single‐clone level. After incubation, ten colonies of each CBE system were randomly selected. Sequencing results showed that no base editing was observed in CBE‐I and CBE‐II and weak editing efficiency was detected in CBE‐III, while CBE‐IV exhibited the highest editing efficiency, and the editing window ranged from C5 to C20 (16 nt) (Figure 4B). To further test the editing efficiency of CBE‐IV, two other genes, *pksE* and *pksG*, were selected as targets for editing. The results showed that CBE‐IV also edited target Cs within a C4 to C22 editing window (≈19 nt) (Figure 4C). The previously reported CBEs generally contain a 5–6 nt editing window (counting the first base close to PAM as position 1) in microbial cells.^[^ ^24^, ^25^, ^26^, ^28^, ^46^ ^]^ Although the editing window was slightly expanded by extending the length of the sgRNA, the editing efficiency decreased accordingly, and Cs close to the PAM sequences still could not be edited. The dBhCas12b‐based CBE has an enlarged editing window (≈19 nt), which is three‐fold wider than the editing window of CBEs currently reported in microbial cells.

To explore the compatibility of dBhCas12b with other types of deaminases, we chose the evolved adenosine deaminase ABE8e^[^ ^37^ ^]^ to construct a dBhCas12b‐based ABE. ABE8e was fused to the N‐terminus of dBhCas12b and integrated into the *lacA* locus of *B. subtilis*, resulting in BS42 (Figure 4D). Three sgRNAs targeting *sigE* were designed, cloned, and inserted into the pHYT vector, resulting in pHYT‐sigE1, pHYT‐sigE2, and pHYT‐sigE3. The three plasmids were transformed into BS42. After incubation, ten colonies of each recombinant strain were selected for sequencing. The sequencing results showed that all of them produced A to G conversions efficiently from A6 to A19 (Figure 4E). The editing window of the dBhCas12b‐based ABE was 2‐fold wider than those of dSpCas9‐ and dLbCas12a‐mediated ABE systems in HEK293T cells.^[^ ^37^ ^]^ These results demonstrated that dBhCas12b is compatible with other types of deaminases and significantly expanded the editing window.

### 2.5 Application of the dBhCas12b‐Based CBE for Strong RBS Screening

Recently, the Base Editor‐Targeted and Template‐free Expression Regulation (BETTER) method was developed for diversifying gene expression in the industrial microorganisms *Corynebacterium glutamicum* and *B. subtilis*.^[^ ^28^ ^]^ BETTER targets a tailored RBS with eight consecutive Cs to construct an RBS library for regulating gene expression. Although BETTER covered eight consecutive Cs, two sgRNAs were necessary because of the limitation of the editing window of CBEs composed of dCas9 or nCas9.

**Figure 5:**
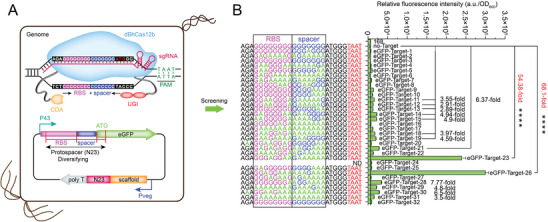
Application of the dBhCas12b‐based CBE for strong RBS screening. A) The general workflow of the dBhCas12b‐based CBE for generating genetic combinations and diversifying gene expression. B) The generated RS sequences and corresponding RIFs of the clones. *B. subtilis* 168 and the tailored RS (fifteen consecutive Gs) were used as controls. RBSs and spacers are shown in magenta and bright blue, respectively. Values and error bars reflect the mean ± s.d. of three independent biological replicates (n = 3). The asterisks indicate significant differences based on a comparison between the experimental group and control group (****P < 0.01, Student's t‐test). PAMs are highlighted in red.

To highlight the advantages of the dBhCas12b‐based CBE, we designed an extreme RBS and spacer fragment (RS) containing fifteen consecutive Gs and examined the editing efficiency with a single sgRNA (**Figure** **5**A). BS41 was used as the starting strain, whose *lacA* locus integrated the major component CDA‐dBhCas12b‐UGI‐UGI. A fluorescent reporter plasmid with the extreme RS and an sgRNA targeting the RS was constructed and transformed into BS41 (Figure 5A). *B. subtilis* 168 served as a negative control, and the fluorescent reporter plasmid without sgRNA served as a positive control. The strains were cultured and induced with 1% xylose until the second generation (12 h per generation) for plating. Thirty‐two clones from the plate were selected for RFI measurement after incubation. The isolated clones exhibited different RFIs, and the values for clones 23 and 26 were 54.38‐ and 68.1‐fold higher than those without sgRNA, respectively (Figure 5B). Sequencing revealed that these clones exhibited different mutations in the RS regions within an ≈17 nt editing window, and clones 23 and 26 exhibited the conserved RBS sequence “GGAGG” (Figure 5B). These results indicated that the CBE could utilize an sgRNA to efficiently target extreme sequence compositions in a wider editing window, making it more robust than BETTER.

### 2.6 The dBhCas12b‐Based CBE Extends Beyond the Protospacer in *Escherichia Coli*

**Figure 6:**
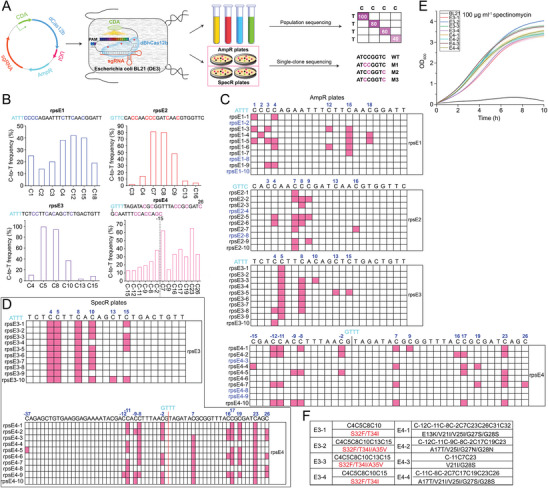
Functional characterization of the dBhCas12b‐based CBE in *E. coli* BL21 (DE3). A) Workflow for base editing identification and verification in *E. coli*. B) Population sequencing of base conversion by the CBE in *rpsE*. Mutated bases at different sites are shown in the corresponding color. PAMs are highlighted in cyan. Source sequencing data can be found in Figure S5 (Supporting Information). C) Base conversion of *rpsE* for clones from ampicillin plates. Partial bases are numbered, and the edited bases are highlighted in bright blue. D) Base conversion in *rpsE* for clones from spectinomycin plates. E) Growth curves of the mutants with 100 µg/ml spectinomycin. The shaded area represents the mean ± s.d. from three biologically independent samples. F) Identification of mutants conferring resistance to spectinomycin by sequencing. The red numbers indicate the substitution of amino acids.

The dBhCas12b‐based CBE exhibited high conversion efficiency and an expanded editing window in *B. subtilis*. Subsequently, we tested whether the CBE could work in other prokaryotes. *E. coli*, as an important industrial microorganism, has been widely used in industrial fermentation and metabolic engineering.^[^ ^47^ ^]^ BEs with expanded editing windows would be useful for chassis design and metabolic engineering of *E. coli*. The evolution of *rpsE* can confer resistance to spectinomycin in RE100 *E. coli*.^[^ ^48^ ^]^ The evolution of *E. coli* BL21 (DE3) for resistance to spectinomycin was carried out by using the dBhCas12b‐based CBE. The CDA‐dBhCas12b‐UGI fragment was cloned and inserted into a temperature‐sensitive pKD46 vector (amp^r^) with an arabinose promoter (P*_araBAD_*), resulting in pKD‐CDA‐dBhCas12b‐UGI. Four sgRNAs targeting *rpsE* (including rpsE1, rpsE2, rpsE3, and rpsE4) were designed and inserted into pKD‐CDA‐dBhCas12b‐UGI (**Figure** **6**A). The four plasmids were transformed into *E. coli* BL21 (DE3). After incubation at 30 °C with 50% arabinose, the conversion efficiency was examined. Population and single‐clone sequencing results showed that base conversion occurred, the conversion efficiency ranged from 2% to 99%, and the editing window targeted by rpsE4 spanned C‐12 to C23 (36 nt), or even C‐15 to C26 (42 nt), extending beyond the entire protospacer (Figure 6B,C; Figure S5, Supporting Information). Compared to the CBEs previously developed in microbial cells,^[^ ^24^, ^25^, ^26^, ^46^ ^]^ the dBhCas12b‐based CBE exhibited high editing efficiency within an ultrawide editing window, approximately eight‐fold wider than those of dCas9 or nCas9‐based CBEs. The Cs close to the PAM (target bases for rpsE1‐3) and the upstream of the protospacer (target bases for rpsE4) could be edited by this tool (Figure 6B,C; Figure S5, Supporting Information).

The clones were spread on plates containing 50 µg mL^−1^ ampicillin and 50 µg mL^−1^ spectinomycin and incubated at 30 °C. All colonies grew normally on plates containing ampicillin, while only those colonies derived from rpsE3 and rpsE4 grew on spectinomycin plates. We selected ten colonies from two targets (rpsE3 and rpsE4) on spectinomycin plates for sequencing. Most of the clones derived from rpsE3 exhibited a 12 nt editing window (C4‐C15), which was similar to the result of population sequencing (Figure 6B). In addition, most of the clones derived from rpsE4 exhibited a 38 nt editing window (C‐11‐C26), and one of the clones exhibited a 64 nt editing window (Figure 6D). Four colonies (E3‐1 to E3‐4 and E4‐1 to E4‐4) derived from rpsE3 and rpsE4 were selected and further incubated on plates containing 100 µg mL^−1^ spectinomycin, and no inhibition was observed. In contrast, the growth of wild‐type *E. coli* BL21 (DE3) was severely inhibited (Figure 6E). Sequencing results showed that all four mutants had corresponding mutations. The conversion of some amino acids (such as V25, G27, and G28) was crucial for conferring spectinomycin resistance to *E. coli*, which is consistent with a previous report.^[^ ^48^ ^]^ Other mutations (such as S32, T34, and A35, red font) conferring spectinomycin resistance to *E. coli* were first discovered (Figure 6F). These results demonstrated that the dBhCas12b‐based CBE could work well in *E. coli* and further expand the editing window. In addition, several spectinomycin‐resistant of *E. coli* strains were successfully obtained.

### 2.7 Comparison of the Editing Performance of CBEs Mediated by Different dCas Proteins

**Figure 7:**
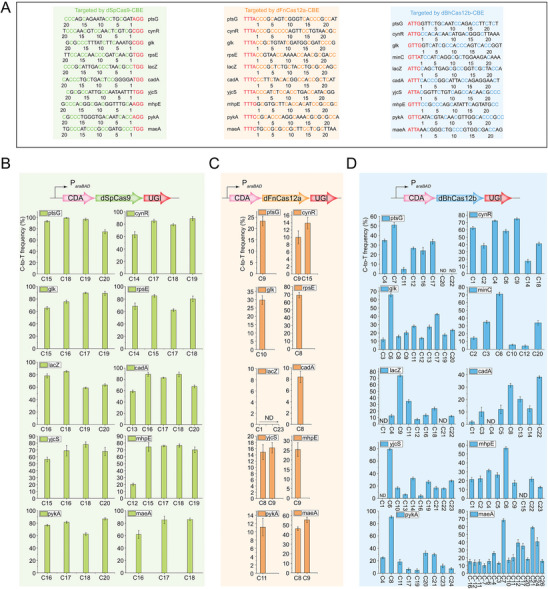
Comparison of editing performance of the different Cas‐mediated CBEs in *E. coli*. A) Genes targeted by different CBEs and their sgRNA sequences. The Cs proximal to the PAM are set as position 1. All potentially editable Cs are highlighted in different colors. PAMs were highlighted in red. B–D) Population sequencing of base conversion by dFnCas12a‐CBE, dSpCas9‐CBE and dBhCas12b‐CBE. Base conversion by dBhCas12b‐CBE exhibited a wider editing window, and the base sequence exhibited on the other side of the PAM (ATTA) is CTTTCCATACCACTGG (*maeA*). All strains were induced by arabinose when the OD_600_ was 0.05. Data are shown as the mean ± s.d. from three independent experiments.

To compare the editing performance of different dCas protein‐mediated CBEs, we selected two other deactivated Cas proteins, dCas9 from *Streptococcus pyogenes* (dSpCas9)^[^ ^5^ ^]^ and dCas12a from *Francisella novicida* U112 (dFnCas12a),^[^ ^49^ ^]^ and constructed the two corresponding CBEs (dSpCas9‐based CBE and dFnCas12a‐based CBE) in *E. coli* JM109. Ten endogenous genes were selected for editing using the three CBEs (**Figure** **7**A). Population sequencing results showed that the dFnCas12a‐based CBE and dSpCas9‐based CBE produced C to T conversion within a narrow editing window (≈4 nt and ≈6 nt), and the bases close to the PAM could not be edited (Figure 7B,C), which is consistent with previous reports.^[^ ^34^ ^]^ In contrast, the dBhCas12b‐based CBE exhibited high editing efficiency (ranging from 3% to 90%) within a wide editing window, and the editing window for *maeA* even expanded from C‐16 to C26 (43 nt) (Figure 7D). These results demonstrated that the dBhCas12b‐based CBE exhibited a surprising editing window compared with CBEs composed of dSpCas9 and dFnCas12a.

It has been reported that a CBE composed of a Cas9 variant (Cas9 with a D10A mutation) has a certain toxicity to *E. coli*, inhibiting cell growth.^[^ ^25^ ^]^ We sought to determine whether the dBhCas12b‐based CBE impacts cell growth. *E. coli* JM109 harboring CDA‐dBhCas12b‐UGI as well as CDA‐dBhCas12b‐UGI and an sgRNA targeting *maeA* was inoculated with an initial OD_600_ of 0.05 and then induced with 50% arabinose. Compared with the control (wild‐type *E. coli* JM109), no inhibition of cell growth was observed in the strains containing CDA‐dBhCas12b‐UGI or CDA‐dBhCas12b‐UGI and an sgRNA targeting *maeA*, indicating that dBhCas12b has no toxicity toward *E. coli* (Figure S6, Supporting Information). In addition, we selected three genes (*cynR*, *maeA*, and *yjcS*) as targets to compare the off‐target effects of CBEs mediated by three Cas proteins (dSpCas9, dFnCas12a, and dBhCas12b), and potential off‐target sites were predicted by Cas‐OFFinder^[^ ^50^ ^]^ (Table S6, Supporting Information). Whole genome sequencing (WGS) showed that no editing activity observed at the predicted off‐target sites, suggesting that the dBhCas12b‐based CBE was a high‐fidelity genome editor.

### 2.8 Application of the CRISPR‐dBhCas12b‐Based CBE for Evolution of the Twin‐Arginine Translocation (Tat) Pathway

**Figure 8:**
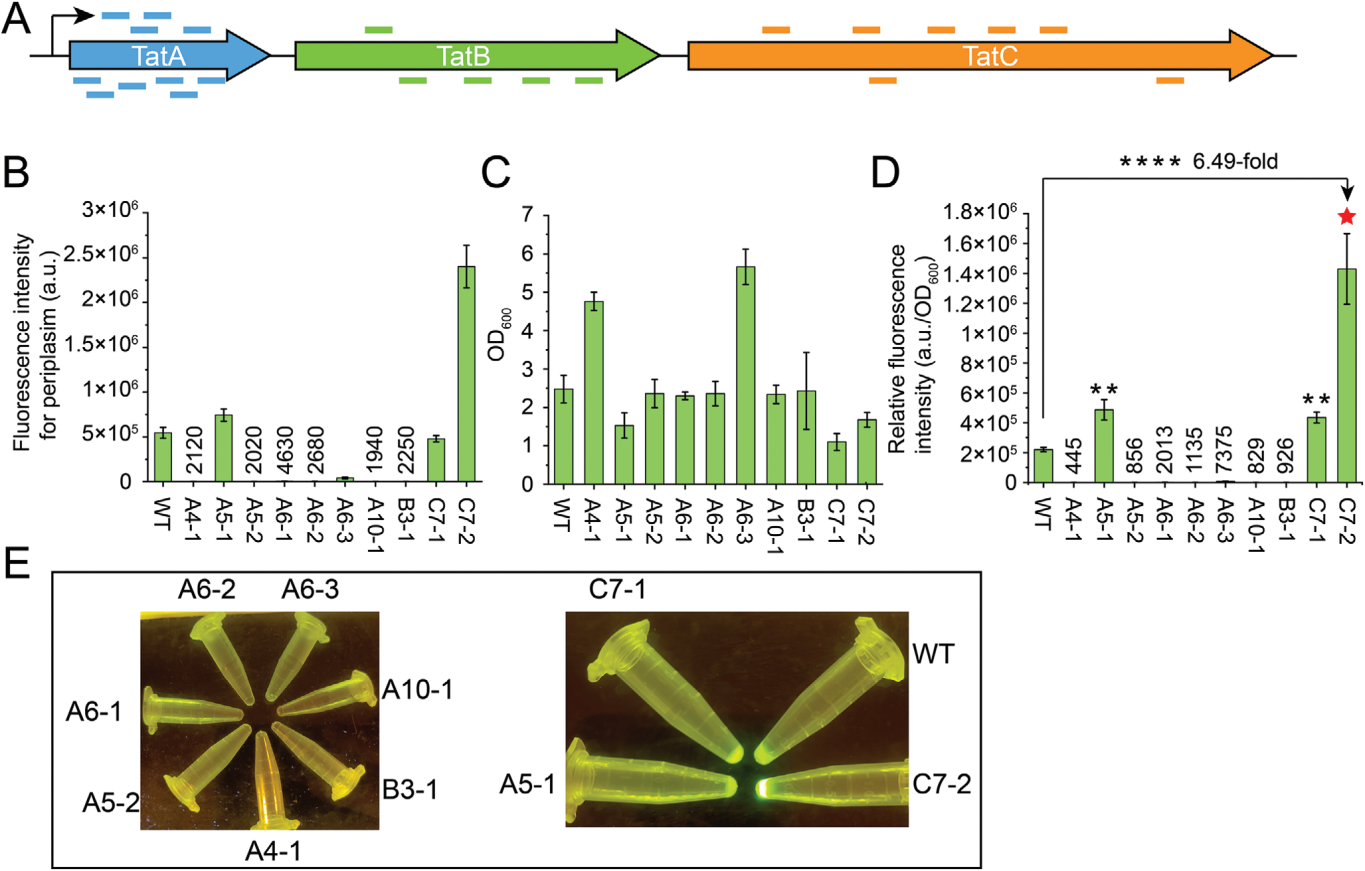
Evolution of the Tat pathway for efficient periplasmic secretion of heterologous proteins. A) Design of a mini‐library for sgRNAs targeting TatABC. Ten sgRNAs targeting TatA, 5 sgRNAs targeting TatB, and 7 sgRNAs targeting TatC. B,C) Total FI of periplasmic sfGFP and cell growth of the recombinant strains. D) RIF of periplasmic sfGFP in the recombinant strains. E) Detection of periplasmic sfGFP from the recombinant strains by a blue light apparatus. Data are shown as the mean ± s.d. from three independent experiments. The asterisks indicate significant differences based on a comparison between the experimental group and control group (**P < 0.05, ****P < 0.01, Student's *t*‐test).

The twin‐arginine translocation (Tat) pathway mediated by Tat translocase (TatABC) is one of the models for heterologous protein delivery into the periplasm of *E. coli*.^[^ ^51^, ^52^, ^53^ ^]^ A functionally improved Tat pathway would enhance protein production;^[^ ^52^ ^]^ thus, TatABC in *E. coli* JM109 was selected for evolution by the dBhCas12b‐based CBE. Twenty‐two sgRNAs (10 sgRNAs targeting TatA, 5 sgRNAs targeting TatB, and 7 sgRNAs targeting TatC) were designed and inserted into pKD46 together with CDA‐dBhCas12b‐UGI (**Figure** **8**A; Figure S7A, Supporting Information). The 22 plasmids were transformed into *E. coli* and induced with 50% arabinose, and then each strain was collected and used for electrotransformation of the reporter plasmid pBAD‐TorA‐sfGFP (containing a Tat‐targeted signal peptide). Five clones from each plate (5 × 22 = 110 clones) were selected for inducing the expression of sfGFP on 96‐well plates. After incubation, the cells were collected and treated to obtain sfGFP secreted into periplasm by the arginine method (see Experimental Section). As a result, ten clones (A4‐1, A5‐1, A5‐2, A6‐1, A6‐2, A6‐3, A10‐1, B3‐1, C7‐1, and C7‐2) with significantly different secretion capacities compared to the wild‐type were isolated (Figure 8B,C). The RFI results showed that the mutant C7‐2 exhibited a 6.49‐fold higher secretion capacity than the wild type (Figure 8D). Meanwhile, the sfGFP isolated from each periplasm was examined by a blue light apparatus, and the mutant C7‐2 exhibited strong fluorescence (Figure 8E). Sequencing results showed that the best mutant, C7‐2, generated a C to T conversion outside the editing window (C26), extending beyond the entire protospacer (Figure S7B, Supporting Information). These results indicate that the dBhCas12b‐based CBE can be used to mutate functional proteins in situ for the construction of higher version chassis.

## 3 Discussion

The use of BEs is a relatively new genome‐editing approach, using components from CRISPR systems together with DNA deaminases to directly install point mutations into cellular DNA.^[^ ^54^ ^]^ This strategy has been widely used as an effective tool for plant breeding^[^ ^37^, ^55^ ^]^ and microbial cell evolution.^[^ ^26^, ^38^ ^]^ However, the current BEs (such as CBEs) developed in microbial cells commonly achieve C to T (or A to G) conversions within an ≈5 nt editing window,^[^ ^24^, ^25^, ^26^, ^46^ ^]^ which allows a maximum of 3 amino acid substitutions. Although the editing window of BEs can be expanded by extending the length of sgRNA in microbial cells, they only have an 8 nt editing window and target bases close to PAM could not be edited. A BE based on nCas9 and a dual‐deaminase has been developed in *B. subtilis*, which simultaneously converts A to G and C to T efficiently, the editing window is only ≈11 nt.^[^ ^38^ ^]^ The evolution of bacterial chassis often requires the accumulation of mutations for functional proteins, and it is difficult to obtain beneficial mutants via a few amino acid substitutions, especially when the key functional regions of the target protein are not clear. In addition, limited by the PAM sequence recognized by Cas proteins, current BEs cannot achieve base conversion covering the entire protospacer. Although current Cas3‐base editing tool is a powerful in vivo mutagenesis platform that enables C to T conversion within an ≈10 kb editing window,^[^ ^56^ ^]^ this platform would generate many unexpected mutations beyond the target gene, lacking gene targeting. Another BE, named CRISPR‐X using dCas9, could generate an editing window of ≈50 nt in mammalian cell,^[^ ^57^ ^]^ it is still unknown whether the platform could work in microbial cell. In fact, we have discovered that the BE constructed using dCas9 exhibited narrow editing window (Figure 7) and very low conversion efficiency in *B. subtilis*,^[^ ^26^ ^]^ indicating the CRISPR‐X maybe not suitable for microbial cell. Thus, it is urgent to develop BEs with expanded editing windows in microbial cell.

Although the factors affecting the editing window are unclear, steric hindrance toward deaminases caused by Cas proteins in the BE system would be a key factor impeding deaminase shifting. Cas12b, which is a newer endonuclease,^[^ ^39^ ^]^ is smaller than Cas9 and Cas12a and has minimal off‐target effects. This feature of Cas12b makes it suitable for constructing BEs with an expanded editing window, as predicted by Porto et al. in their review.^[^ ^45^ ^]^ Therefore, the construction of Cas12b‐based BEs was carried out, and a dBhCas12b‐based CBE converting C to T was successfully obtained, which exhibited high conversion efficiency and an expanded editing window, as we predicted. dBhCas12b exhibited fine compatibility with other deaminases, and the dBhCas12b‐based ABE using adenosine deaminase for A to G conversion also exhibited high conversion efficiency and an expanded editing window. The excellent editing performance of the dBhCas12b‐based CBE and dBhCas12b‐based ABE can efficiently produce a mutant library with much greater diversity in vivo, with promising applications in protein and cell evolution. Additionally, our previous studies have shown that the regulation of deaminase‐Cas fusion expression level can fine‐tune the mutation frequencies of the target bases within an editable window by changing inducer concentration and regulating cell generation.^[^ ^26^, ^38^ ^]^ Thus, mutation diversification will further increase by regulating the expression level of the dBhCas12b‐based BEs and cell generation.

The dBhCas12b‐based BE constructed here not only worked well in *B. subtilis* but was also suitable for *E. coli*, a gram‐negative bacterium, indicating that it could be applied in other microbial cells. It exhibited a wider editing window of up to 43 nt, covering the entire protospacer or more (Figure 6). This is the first time a BE has exhibited full‐coverage editing of the protospacer. The UGI used for the dBhCas12b‐based CBE in *E. coli* had only one copy, not two copies as used for *B. subtilis*. In fact, both one copy and two copies of the UGI used for the dBhCas12b‐based CBE in *E. coli* exhibited the same conversion efficiency and editing window (data not shown). This may be due to the stronger DNA repair ability of *B. subtilis* than that of *E. coli*; one copy of the UGI in the dBhCas12b‐based CBE of *E. coli* could sufficiently inhibit the reverse mutation of T to C. The application of BEs is limited by the narrow editing window; editing must be performed repeatedly by using many more sgRNAs, and obtaining the expected strain is inefficient. Several studies aiming to expand the BE editing window have been reported. A CBE composed of nCas9 and human APOBEC3A (hA3A) developed in plant cells converts C to T efficiently in wheat, rice, and potato within a 17 nt editing window,^[^ ^58^ ^]^ and a potent miniCBE based on dUn1Cas12f1 developed for correcting pathogenic mutations converts C to T within an editing window (C3‐C20).^[^ ^59^ ^]^ Although the editing window has been expanded in these BEs, the target bases close to the PAM could not be edited. In contrast, the dBhCas12b‐based BE not only edited the bases close to the PAM but also edited the upstream bases of the protospacer (Figure 6), exhibiting the widest editing window among the reported BEs at present.^[^ ^13^, ^15^, ^60^ ^]^

Off‐target editing activity is one of the key factors for gene editing. To this end, the off‐target effects of CBE composed of three Cas proteins were further evaluated, potential off‐target sites were predicted by Cas‐OFFinder.^[^ ^50^ ^]^ WGS showed that no editing activity was detected at the predicted off‐target sites (Table S6, Supporting Information), suggesting that the dBhCas12b‐based BE system had almost undetectable off‐target editing activity, which is consistent with a previous report showing that Cas12b has minimal off‐target effects.^[^ ^42^ ^]^

As a successful proof of chassis cell evolution using the dBhCas12b‐based CBE, one *E. coli* strain BL21 (DE3) with resistance to spectinomycin and another *E. coli* strain JM109 with a high‐performance Tat pathway were successfully obtained efficiently. Compared with BEs based on dCas9 or nCas9, which generally have an editing window of 5–6 nt, corresponding to a maximum of 2–3 amino acids (excluding synonymous mutations due to codon wobble), the dBhCas12b‐based CBE is capable of inducing up to 8 amino acid conversions, which is several orders of magnitude greater than those BEs theoretically. It could be recognized as a new‐generation BE with an expanded editing window.

## 4 Experimental Section

### Strains and Culture Conditions

The strains used in this study are listed in Table S1 (Supporting Information). The *E. coli* strain JM109 that was used for general cloning was cultivated aerobically at 37 °C in Luria–Bertani (LB) broth. For base editing, the *E. coli* strains JM109 and BL21 (DE3) were cultivated aerobically at 30 °C in LB broth. Ampicillin (50 µg mL^−1^) or kanamycin (50 µg mL^−1^) was added to the medium as needed. *B. subtilis* strain 168 and its derivatives were cultivated aerobically at 37 °C in LB broth. Kanamycin (50 µg mL^−1^), tetracycline (15 µg mL^−1^), chloramphenicol (5 µg mL^−1^), or spectinomycin (50 µg mL^−1^) was added as needed.

### Plasmid Construction

The plasmids used in this study are listed in Table S2 (Supporting Information). The primers used for plasmid construction are listed in Table S3 (Supporting Information). Plasmids were constructed via homologous recombination. Homologous recombination was conducted using the 2× MultiF Seamless Assembly Kit (ABclonal, Nanjing, China).

### Design of the Gene Expression Cassette

To construct an sgRNA targeting a tailored RBS and spacer (RS), the third lysine of eGFP was mutated to asparagine (K3N), thus obtaining a PAM (ATTA) recognized by dBhCas12b. Then, the original RS (AAAGGAGGAAAAAAA) was replaced with the tailored RS (GGGGGGGGGGGGGGG) using reverse PCR. eGFP was expressed using the strong constitutive promoter P43.

### Generation of Genetic Variants with Base Editing

The pB‐P43‐mRS‐eGFPsg plasmid expressing the sgRNA targeting the tailored RS element was transformed into BS41. The resulting transformants were iteratively cultivated (two generations, 12 h per generation) in LB medium supplemented with kanamycin and 1% xylose for base editing. The edited culture was diluted and plated onto LB plates supplemented with kanamycin. The resulting clones were used as templates for colony PCR to amplify the RS region, and the PCR products were sequenced to identify the expected mutation.

### Chromosomal Integration

A marker‐free genome editing approach was used to perform gene integration for overexpression in *B. subtilis* as previously reported. The integration of the foreign gene CDA‐dBhCas12b‐UGI‐UGI was taken as an example (the integration of other foreign genes was similar to that of CDA‐dBhCas12b‐UGI‐UGI). Specifically, the integration vector pAX‐CDA‐dBhCas12b‐UGI‐UGI was used as a template for PCR to amplify the P*_xylA_*‐CDA‐dBhCas12b‐UGI‐UGI expression cassette along with the *lacA* homologous arms on the flanks. PCR products were purified and transformed into *B. subtilis* 168. Theoretically, the foreign gene P*_xylA_*‐CDA‐dBhCas12b‐UGI‐UGI could be integrated into the *lacA* locus of *B. subtilis* through intracellular recombinase‐mediated homologous recombination. To further determine whether the foreign gene was correctly integrated into the target locus, customized primer pairs were needed to amplify the target locus. All foreign gene integration vectors derived from pAX01 and pDGT are listed in Table S2 (Supporting Information).

### Repression of Gene Transcription in *B. subtilis*

For the repression of transcriptional elongation for eGFP expression (taking BS2 as an example), a fluorescence‐based reporter plasmid, pB‐P43‐eGFP, was transformed into BS2, whose genomic *lacA* and *amyE* loci were integrated with dBhCas12b and eGFP‐targeting sgRNA, respectively. The resulting recombinant strain was cultured overnight in LB medium containing 1% xylose and kanamycin. The culture was transferred into 96‐well black‐walled plates and analyzed using a PerkinElmer EnSpire 2300 Multimode Plate Reader (excitation at 485 nm and emission at 528 nm).

### Gene Deletion for *B. subtilis* and Base Editing for *B. subtilis and E. coli*

The BhCas12b‐based CRISPR system was used for the deletion of target genes in *B. subtilis* (taking *sacA* as an example). The plasmid (the detailed plasmid construction process is described in the Supporting Information) was transformed into *B. subtilis*, and the subsequent recombinant strain was cultured overnight in liquid LB medium for gene deletion. Then, the culture was diluted and spread on plates containing chloramphenicol, and the resulting clones were used to further confirm whether the target gene was deleted through colony PCR. The gene deletion process of CRISPR‐BhCas12b was the same as that of CRISPR‐AaCas12b.

Base editing in *B. subtilis* (taking *pksA* as an example) was performed as follows. The plasmid targeting *pksA* (the detailed plasmid construction process is described in the Supporting Information) was transformed into the recombinant strains BS38, BS39, BS40, and BS41, whose *lacA* loci were inserted with the fragments dBhCas12b‐CDA, CDA‐dBhCas12b, CDA‐dBhCas12b‐UGI, and CDA‐dBhCas12b‐UGI‐UGI, respectively. BS38, BS39, BS40, and BS41 with plasmids targeting *pksA* were cultured in 1% xylose, and the edited culture was diluted and spread on LB plates containing tetracycline. The resulting clones were used as templates to amplify the position of the expected mutation, and the PCR products were further sequenced.

Base editing in *E. coli* (taking *rpsE* as an example) was performed as follows. The *rpsE*‐targeting plasmid was transformed into *E. coli*, and the resulting recombinant strains were cultured and induced by 50% arabinose at 30 °C. The induced culture was partially diluted and spread on LB plates containing ampicillin to investigate editing efficiency at the single‐clone level, and the other part was used as a template to amplify the position of the expected mutation to investigate editing efficiency at the population level. The raw data for population sequencing were analyzed by the previously described method. All sgRNA sequences are listed in Table S4 (Supporting Information).

### Plasmid Curing

*B. subtilis* harboring the gene deletion plasmid was inoculated into LB medium containing 0.005% sodium dodecyl sulfate (SDS) without antibiotics and incubated at 37 °C and 200 rpm for ≈20 h for plasmid curing. The temperature‐sensitive plasmid pKD46 was used to construct BEs in *E. coli* JM109 or BL21 (DE3). After editing at 30 °C, *E. coli* containing pKD series plasmids was cultured at 42 °C for ≈12 h for plasmid curing.

### Molecular Docking of dBhCas12b and sgRNA

RosettaFoldNA ^[^ ^61^ ^]^(https://github.com/uw‐ipd/RoseTTAFold2NA) was used to construct the model of the BhCas12b‐binding RNA complex, which served as a structural reference for the design of dBhCas12b. The initial complex structure was improved by two rounds of optimization with the FastRelax module^[^ ^62^ ^]^ of Rosetta 2021.16. In the first round of optimization, 40 results that were optimized with internal coordinates were output and then scored and ranked according to the ref2015_cart weights. The best structure was used for the second round of optimization, and 200 structures optimized by Cartesian space sampling methods were generated and then scored and ranked according to the ref2015_cart weights to determine the optimal structure.

### Extraction of Periplasmic sfGFP

Cultured cells were centrifuged at 3500–4500 rpm for 10 min at 4 °C, the supernatant was discarded, and the cells were washed once with PBS. Then, 40 mm (pH 9.0) arginine solution was added to the cells at a ratio of 1:40 (v/v), aspirated and mixed well, and placed in a 4 °C ice bath for 30 min. The treated cells were centrifuged at 4500 rpm for 10 min at 4 °C, and the resulting supernatant was the periplasmic protein fraction.

### Whole Genome Sequencing and Off‐Target Analysis

The culture of the base‐edited cells was prepared. Approximately, 109 cells were used for extraction of genomic DNAs. NEBNext Ultra DNA Library Prep Kit was used to convert the amplicon into indexed libraries for WGS on the Illumina platform. Library construction and sequencing were performed by GENEWIZ (Suzhou, China). Approximately 20 000 000–30 000 000 reads per sample were analyzed. Base‐substitution frequencies were calculated by dividing base‐substitution reads by total reads. For WGS off‐target analysis, off‐target sites of selected target loci were analyzed by Cas‐OFFinder.^[^ ^50^ ^]^ All similar sequences of selected target loci were chosen as predicted off‐target sites (Table S6, Supporting Information).

### Statistical Analysis

Values and error bars reflect the mean ±s.d. of three biological replicates (*n* = 3). GraphPad Prism 9.0.0 was used for statistical analysis. Data between two groups were assessed using Student's t‐test. The results were considered statistically significant at ^**^ *p* < 0.05 and ^****^ *p* < 0.01.

## Conflict of Interest

The authors declare no conflict of interest.

## Author Contributions

H.W. and C.W. performed all of the experiments with help from all authors. H.W. and H.L. wrote the manuscript with input from all authors. S.F., W.Y., and L.Z. provided technical advice. H.W. and Z.Z. conceived the project.

## Supporting information

Supporting Information

## Data Availability

The data that support the findings of this study are available from the corresponding author upon reasonable request.
