# Supplementary material for: A New‐Generation Base Editor with an Expanded Editing Window for Microbial Cell Evolution In Vivo Based on CRISPR‒Cas12b Engineering
Source: Adv Sci (Weinh). 2024 Apr 11;11(22):2309767. doi: 10.1002/advs.202309767 (PMC11165516; doi:10.1002/advs.202309767)
Supplement: Supplementary file 1 — Supporting Information [file ADVS-11-2309767-s001.pdf]

ADVANCED SCIENCE
Open Access

Supporting Information

for *Adv. Sci.*, DOI 10.1002/advs.202309767

A New-Generation Base Editor with an Expanded Editing Window for Microbial Cell Evolution In Vivo Based on CRISPR–Cas12b Engineering

Wenliang Hao, Wenjing Cui, Zhongmei Liu, Feiya Suo, Yaokang Wu, Laichuang Han and Zhemin Zhou**

A New-Generation Base Editor with An Expanded Editing Window for Microbial Cell Evolution *In vivo* Based on CRISPR–Cas12b Engineering

Wenliang Hao, Wenjing Cui, Zhongmei Liu, Feiya Suo, Yaokang Wu, Laichuang Han*, Zhemin Zhou*

Supporting Information

Table S1. The strains used in this study.

Table S2. The plasmids used in this study.

Table S3. The primers used for gene cloning in this study.

Table S4. The promoter sequences used in this study.

Table S5. The protospacer sequences used in this study.

Table S6. The off-target analysis of dSpCas9-CBE, dFnCas12a-CBE, and dBhCas12b-CBE in *E. coli*.

Figure S1. Investigation of the *sacA* deletion efficiency for different variants of BhCas12b.

Figure S2. The relevant elements of eGFP expression cassette and the positions of sgRNA in the eGFP coding region.

Figure S3. Analysis of cell density and total fluorescence intensity using dBhCas12b-based CRISPRi system.

Figure S4. Analysis of transcription initiation repression by dBhCas12b-based CRISPRi system.

Figure S5. Sample image output of the sequencing data for the editing of *rpsE* from the BEAT analysis.

Figure S6. The impact of dBhCas12b on the growth of *E. coli*.

Figure S7. Sequencing results for the Tat translocase mutants isolated from the mutant library.

Supplementary Method. Construction of plasmids.

Supplementary Sequences. Sequence of key genetic parts.

Table S1. The strains used in this study

Strains	Description	References or sources
E. coli JM109	*recA*1, *supE*44 *endA*1 *hsdR*17 (r^-k,m$^+$k) *gyrA*96 *relA*1 thi (*lac-proAB*) F'[*traD*36 *proAB*$^+$ *lacI*^{q} *lac*ZΔM15]	Lab stock
E. coli BL21 (DE3)	F$^-$, *ompT, hsdS* (rBB$^-$, mB$^-$), *gal, dcm*	Lab stock
B. subtilis 168	*trpC*2	Lab stock
BS1	Derived from *B. subtilis* 168, *lacA*::P_{xylA}-dBhCas12b	This study
BS2	Derived from BS1, *amyE*::P_{veg}-sgRNA (G1)	This study
BS3	Derived from BS1, *amyE*::P_{veg}-sgRNA (G2)	This study
BS4	Derived from BS1, *amyE*::P_{veg}-sgRNA (G3)	This study
BS5	Derived from BS1, *amyE*::P_{veg}-sgRNA (G4)	This study
BS6	Derived from BS1, *amyE*::P_{veg}-sgRNA (G5)	This study
BS7	Derived from BS1, *amyE*::P_{veg}-sgRNA (G6)	This study
BS8	Derived from BS1, *amyE*::P_{veg}-sgRNA (G7)	This study
BS9	Derived from BS1, *amyE*::P_{veg}-sgRNA (G8)	This study
BS10	Derived from BS1, *amyE*::P_{veg}-sgRNA (G9)	This study
BS11	Derived from BS1, *amyE*::P_{veg}-sgRNA (G10)	This study
BS12	Derived from BS1, *amyE*::P_{veg}-sgRNA (G11)	This study
BS13	Derived from BS1, *amyE*::P_{veg}-sgRNA (G12)	This study

BS14	Derived from BS1, *amyE*::P_{veg}-sgRNA (G13)	This study
BS15	Derived from BS1, *amyE*::P_{veg}-sgRNA (G14)	This study
BS16	Derived from BS1, *amyE*::P_{veg}-sgRNA (G15)	This study
BS17	Derived from BS1, *amyE*::P_{veg}-sgRNA (P43-1)	This study
BS18	Derived from BS1, *amyE*::P_{veg}-sgRNA (P43-2)	This study
BS19	Derived from BS1, *amyE*::P_{veg}-sgRNA (P43-3)	This study
BS20	Derived from BS1, *amyE*::P_{veg}-sgRNA (P43-4)	This study
BS21	Derived from BS1, *amyE*::P_{veg}-sgRNA (relA-1)	This study
BS22	Derived from BS1, *amyE*::P_{veg}-sgRNA (relA-2)	This study
BS23	Derived from BS1, *amyE*::P_{veg}-sgRNA (relA-3)	This study
BS24	Derived from BS1, *amyE*::P_{veg}-sgRNA (relA-4)	This study
BS25	Derived from BS1, *amyE*::P_{veg}-sgRNA (ylbP-1)	This study
BS26	Derived from BS1, *amyE*::P_{veg}-sgRNA (ylbP-2)	This study
BS27	Derived from BS1, *amyE*::P_{veg}-sgRNA (ylbP-3)	This study
BS28	Derived from BS1, *amyE*::P_{veg}-sgRNA (ylbP-4)	This study
BS29	Derived from BS1, *amyE*::P_{veg}-sgRNA (spoVG-1)	This study
BS30	Derived from BS1, *amyE*::P_{veg}-sgRNA (spoVG-2)	This study
BS31	Derived from BS1, *amyE*::P_{veg}-sgRNA (spoVG-3)	This study
BS32	Derived from BS1, *amyE*::P_{veg}-sgRNA (spoVG-4)	This study
BS33	Derived from BS1, *amyE*::P_{veg}-sgRNA (rpoB-1)	This study
BS34	Derived from BS1, *amyE*::P_{veg}-sgRNA (rpoB-2)	This study
BS35	Derived from BS1, *amyE*::P_{veg}-sgRNA (rpoB-3)	This study

BS36	Derived from BS1, *amyE*::P_{veg}-sgRNA (sigW-1)	This study
BS37	Derived from BS1, *amyE*::P_{veg}-sgRNA (sigW-2)	This study
BS38	Derived from *B. subtilis* 168, *lacA*::P_{xylA}-dBhCas12b-CDA	This study
BS39	Derived from *B. subtilis* 168, *lacA*::P_{xylA}-CDA-dBhCas12b	This study
BS40	Derived from *B. subtilis* 168, *lacA*::P_{xylA}-CDA-dBhCas12b-UGI	This study
BS41	Derived from *B. subtilis* 168, *lacA*::P_{xylA}-CDA-dBhCas12b-UGI-UGI	This study
BS42	Derived from *B. subtilis* 168, *lacA*::P_{xylA}-ABE8e-dBhCas12b	This study

Table S2. Primers used in this study

Primers	Sequences (5'-3')
BhCas12b-F	ttgcgttgcgcttacatggactgcttggagga
BhCas12b-R	ggaggaaaaaaaatggctacgagaagcttcatt
BhCas12b-b-F	ttctcgtagccattttttttcctccttttctcgaggg
BhCas12b-b-R	agcagtccatgtaagcgcaacgcaattaatgtga
sacABhsg-F	tatcttatcacttattaacgttgatataatttaaattttatttgacaaaaa
sacABhsg-R	gcagacaaagaaaaaaaatggacgattttggatacatg
sacABhsg-b-F	ccattttttttctttgtctgcaactgaaaagtt
sacABhsg-b-R	atcaacgttaataagtgataagataaaaaatttttcacgcttac
pHT-aprEsg-F	actacagcgctgaaaacagtcgttgttttttttctttgtctgcaactgaaaagtt
pHT-aprEsg-R	aaaaacaacgactgttttcagcgctgtagtgctaatgcctcgtaagagacat
pHT-aprEHA-F	gaaaagtttataattatgggccacgaaatggg
pHT-aprEHA-R	gttccaggtaaggccgatattggttaaacagcgg
pHT-aprEHA-F	aaccaatatcggccttacctggaacaaatggttg
pHT-aprEHA-R	cgtggcccataattataaacttttcagttgcagacaaagga
AaCas12b-F	ttgcgttgcgcttaaatatcgcccgtgttttcg

AaCas12b-R	aaggaggaaaaaaaatggccgtcaagtccatgaa
AaCas12b-b-F	ttgacggccatttttttcctccttttctcgaggg
AaCas12b-b-R	cgggcgatatttaagcgcaacgcaattaatgtga
sacAAasg-F	tcttatcacttattaacgttgatataatttaaattttatttgacaaa
sacAAasg-R	ttgcagacaaagaaaaaaaaagcctgaactgaagca
sacAAasg-b-F	aggcttttttttctttgtctgcaactgaaaagtttatac
sacAAasg-b-R	atatcaacgttaataagtgataagataaaaaatttttcacgctt
pAX-dBhCas12b-F	gaaatgggatccatggctacgagaagcttcattctg
pAX-dBhCas12b-R	tccttactcgagttacatggactgcttggaggaatc
pAX-dBhCas12b-b-F	agcagtccatgtaactcgagtaaggatctccaggcat
pAX-dBhCas12b-b-R	cttctcgtagccatggatcccatttcccccttgattttta
BhsgRNA-F	gacgggatccttattaacgttgatataatttaaattttatttgacaaaaatg
BhsgRNA-R	gcccaagcttaaaaaaaagaaaaaaaatgggtaagggag
BhsgRNA-b-F	tcttttttttaagcttgggcttaattaattaagactc
BhsgRNA-b-R	caacgttaataaggatcccgtcgacgc
G1-F	tcccttacccattttttttcttttttttaagcttgggcttaattaattaag
G1-R	gaaaaaaaatgggtaagggagaagtgctaatgcctcgtaagag
G2-F	aagaattgggacaactccagtttttttaagcttgggcttaattaattaag
G2-R	ctggagttgtcccaattcttgttgtgctaatgcctcgtaagag
G3-F	ttgaattagatggtgatgttttttttttaagcttgggcttaattaattaag
G3-R	aacatcaccatctaattcaacaagtgctaatgcctcgtaagag
G4-F	ggcacaaattttctgtcagtttttttttaagcttgggcttaattaattaag
G4-R	actgacagaaaatttgtgcccatgtgctaatgcctcgtaagag
G5-F	gtcagtggagagggtgaaggtttttttaagcttgggcttaattaattaag
G5-R	ccttcaccctctccactgacagagtgctaatgcctcgtaagag
G6-F	aaggaacaggcagctttccatttttttaagcttgggcttaattaattaag
G6-R	tggaaagctgcctgttccttggcgtgctaatgcctcgtaagag
G7-F	ggccaacacttgtcactacttttttttaagcttgggcttaattaattaag
G7-R	agtagtgacaagtgttggccaaggtgctaatgcctcgtaagag
G8-F	taagctcgattctgttgacgtttttttaagcttgggcttaattaattaag
G8-R	cgtcaacagaatcgagcttaagggtgctaatgcctcgtaagag
G9-F	ggaggacggaaacatcctcgtttttttaagcttgggcttaattaattaag
G9-R	cgaggatgtttccgtcctccttggtgctaatgcctcgtaagag
G10-F	ctaattttgaagttaacttttttttttaagcttgggcttaattaattaag
G10-R	aaagttaacttcaaaattagacagtgctaatgcctcgtaagag
G11-F	tcaaaattagacacaacatttttttttaagcttgggcttaattaattaag
G11-R	aatgttgtgtctaattttgaagtgtgctaatgcctcgtaagag
G12-F	tgataatggtctgctagttgtttttttaagcttgggcttaattaattaag
G12-R	caactagcagaccattatcaacagtgctaatgcctcgtaagag

G13-F	acaaaatactccaattggcgtttttttttaagcttgggcttaattaattaag
G13-R	cgccaattggagtattttgttgagtgctaatgcctcgtaagag
G14-F	ggacaggtaatggttgtctgtttttttttaagcttgggcttaattaattaag
G14-R	cagacaaccattacctgtccacagtgctaatgcctcgtaagag
G15-F	gtccacacaatctgcccttttttttttttaagcttgggcttaattaattaag
G15-R	aaagggcagattgtgtggacagggtgctaatgcctcgtaagag
P43-1-F	tttttgccgtgatttcgtgttttttttttaagcttgggcttaattaattaag
P43-1-R	acacgaaatcacggcaaaaacgcgtgctaatgcctcgtaagag
P43-2-F	aaaatgtaaaataaatgtaattttttttttaagcttgggcttaattaattaag
P43-2-R	ttacatttattttacatttttaggtgctaatgcctcgtaagag
P43-3-F	gaaatgggcgtgaaaaaaagttttttttaagcttgggcttaattaattaag
P43-3-R	cttttttttcacgcccatttctaagtgctaatgcctcgtaagag
P43-4-F	ataatcgcgcgcttttttttcttttttttaagcttgggcttaattaattaag
P43-4-R	gaaaaaaagcgcgcgattatgtagtgctaatgcctcgtaagag
ylbP-1-F	aaatccaaatatttaaacttttttttttttaagcttgggcttaattaattaag
ylbP-1-R	aagtttaaatatttggatttttttgtgctaatgcctcgtaagag
ylbP-2-F	tttttttaaataaagcgtttatttttttttaagcttgggcttaattaattaag
ylbP-2-R	taaacgctttatttaaaaaaatccgtgctaatgcctcgtaagag
ylbP-3-F	aaataaagcgtttacaatattttttttttaagcttgggcttaattaattaag
ylbP-3-R	atattgtaaacgctttatttaaagtgctaatgcctcgtaagag
ylbP-4-F	atatatgtagaaacaacaactttttttttaagcttgggcttaattaattaag
ylbP-4-R	gttgttgtttctacatatattgtgtgctaatgcctcgtaagag
rpoB-1-F	tagttaaaataccgagtcaatttttttttaagcttgggcttaattaattaag
rpoB-1-R	ttgactcggtattttaactatgtgtgctaatgcctcgtaagag
rpoB-2-F	ctatgttaatattgtaaaattttttttttaagcttgggcttaattaattaag
rpoB-2-R	attttacaatattaacatagttagtgctaatgcctcgtaagag
rpoB-3-F	ttttacaatattaacatagttttttttttaagcttgggcttaattaattaag
rpoB-3-R	actatgttaatattgtaaaatgcgtgctaatgcctcgtaagag
relA-1-F	tgcatttattttatataatattttttttttaagcttgggcttaattaattaag
relA-1-R	tattatataaaataaatgcaaaagtgctaatgcctcgtaagag
relA-2-F	aaaataaatgcaaaacaatattttttttttaagcttgggcttaattaattaag
relA-2-R	tattgttttgcatttattttatagtgctaatgcctcgtaagag
relA-3-F	cttctgctctttacacctcgttttttttttaagcttgggcttaattaattaag
relA-3-R	cgaggtgtaaagagcagaagttcgtgctaatgcctcgtaagag
relA-4-F	tatttgaacttctgctctttttttttttttaagcttgggcttaattaattaag
relA-4-R	aaagagcagaagttcaaatagccgtgctaatgcctcgtaagag
spoVG-1-F	aaacgagcaggatttcagaattttttttttaagcttgggcttaattaattaag
spoVG-1-R	ttctgaaatcctgctcgtttttagtgctaatgcctcgtaagag
spoVG-2-F	gatttttttctgaaatcctgctttttttttaagcttgggcttaattaattaag
spoVG-2-R	gcaggatttcagaaaaaaatcgtggtgctaatgcctcgtaagag
spoVG-3-F	aaaaaaatcgtggaattgatattttttttttaagcttgggcttaattaattaag
spoVG-3-R	tatcaattccacgatttttttctggtgctaatgcctcgtaagag
spoVG-4-F	cactaatgcttttatcctcgtttttttttaagcttgggcttaattaattaag

spoVG-4-R	cgaggataaaagcattagtgtatgtgctaatgcctcgtaagag
sigW-1-F	aaaaaaattgaaaccttttgtttttttaagcttgggcttaattaattaag
sigW-1-R	caaaaggtttcaattttttatagtgctaatgcctcgtaagag
sigW-2-F	ccttttgaaacgaagctcgtttttttttaagcttgggcttaattaattaag
sigW-2-R	acgagcttcgtttcaaaaggtttgtgctaatgcctcgtaagag
mRBSsp-F	ggggggggggggggatgggtaatggagaagaacttttcactggagtt
mRBSsp-R	ttacccatcccccccccccccctctcgagggtaccgctatca
mRBSsp-sg-F	gagcaaaaccgtcctcgccgaaaatgacc
mRBSsp-sg-R	cagcaaagggggatagcaccacatagcagacc
mRBSsp-sg-b-F	gtggtgctatccccctttgctgaggtgg
mRBSsp-sg-b-R	cggcgaggacggttttgctctcgtgctc
pAX-cCDA-F	gaaatgggatccatggctacgagaagcttcattc
pAX-cCDA-R	aagctgcagaacccatggactgcttggaggaatc
pAX-cCDA-b-F	caagcagtccatgggttctgcagcttctagaatg
pAX-cCDA-b-R	tcgtagccatggatcccatttccccctttgatttttag
pAX-nCDA-F	ggaaatgggatccatgacagatgccgaatacgttc
pAX-nCDA-R	tcgtagccattctagaagctgcagaaccgac
pAX-nCDA-b-F	agcttctagaatggctacgagaagcttcattctg
pAX-nCDA-b-R	cggcatctgtcatggatcccatttccccctttgattttta
pAX-UGI-F	tccaagcagtccatgggaccgaagaagaagcgc
pAX-UGI-R	gatccttactcgagttaaagcattttgatcttattctcgcc
pAX-UGI-b-F	caaaatgctttaactcgagtaaggatctccagg
pAX-UGI-b-R	cttcttcggtcccatggactgcttggaggaatc
pAX-2UGI-F	ctcctggaaccagtgagtctgcaactccagagtctatgacgaatcttagcgacatcatc
pAX-2UGI-R	tccttactcgagttaaagcattttgatcttattctcgcc
pAX-2UGI-F	caaaatgctttaactcgagtaaggatctccagg
pAX-2UGI-R	gcagactcactggttccaggagtttcagaacctgaaagcattttgatcttattctcgcc
pksA-F	ttgctctagattattaacgttgatataatttaaattttatttgacaaaaatgg
pksA-R	tgtgctgaagaaaaaaaagggatggagggag
pksA-b-F	ccctttttttcttcagcacaattccaagaaaaac
pksA-b-R	cgttaataatctagagcaagcttgggcaaag
pksC-F	ttgctctagattattaacgttgatataatttaaattttatttgacaaaaatgg
pksC-R	catcagctcgcctttttttcttcagcaca
pksC-b-F	cctttttttcttcagcacaattccaagaaaaacac
pksC-b-R	cgttaataatctagagcaagcttgggcaaag
pKD-CDA-F	gttataaaaaatgacagatgccgaatacgttc
pKD-CDA-R	cagccggatcttaaagcattttgatcttattctcgcc
pKD-CDA-b-F	caaaatgctttaagatccggctgctaacaaag
pKD-CDA-b-R	catctgtcattttttataacctccttagagctcgaattc
pKD-Bhsg-F	ttcggattatttattaacgttgatataatttaaattttatttgacaaaaatg
pKD-Bhsg-R	ctgtcacgggaaaaaaaaaatccgttgaagaaattctg
pKD-Bhsg-b-F	ggattttttttttcccgtgacaggtcattcaga
pKD-Bhsg-b-R	acgttaataaataatccgaagtggtcagactg

rpsE1-F	ccccagaatttcttcaacggattttttttttcccgtgacaggtc
rpsE1-R	aatccgttgaagaaattctgggggtgctaatgcctcgtaagag
rpsE2-F	caccaacccgatcaacgtggttcttttttttcccgtgacaggtc
rpsE2-R	gaaccacgttgatcgggttggtggtgctaatgcctcgtaagag
rpsE3-F	tctccttcacagctctgactgtttttttttcccgtgacaggtc
rpsE3-R	aacagtcagagctgtgaaggagagtgctaatgcctcgtaagag
rpsE4-F	tagatacgcggtttaccgcgatcttttttttcccgtgacaggtc
rpsE4-R	gatcgcggtaaaccgcgtatctagtgctaatgcctcgtaagag
BhcadA-F	cacccggcattaccagaggaacttttttttcccgtgacaggtc
BhcadA-R	agttcctctggtaatgccgggtggtgctaatgcctcgtaagag
BhcynR-F	ccacacaacatgacgggcttaaattttttttcccgtgacaggtc
BhcynR-R	tttaagcccgtcatgttgtgtgggtgctaatgcctcgtaagag
Bhglk-F	gtcatcgccacccagtcaccggttttttttcccgtgacaggtc
Bhglk-R	accggtgactgggtggcgatgacgtgctaatgcctcgtaagag
BhlacZ-F	cagctgagcgccggtcgctaccattttttttcccgtgacaggtcattc
BhlacZ-R	tggtagcgaccggcgctcagctggtgctaatgcctcgtaagagacatc
BhmaeA-F	aacgggctgcccgtggcgaccagttttttttcccgtgacaggtc
BhmaeA-R	ctggtcgccacgggcagcccgttgtgctaatgcctcgtaagag
BhmhpE-F	ccgcccagcatattcagtatgccttttttttcccgtgacaggtc
BhmhpE-R	ggcatactgaatatgctgggcgggtgctaatgcctcgtaagag
BhminC-F	tccatcaggcgctggaagacaaattttttttcccgtgacaggtc
BhminC-R	tttgtcttccagcgcctgatggagtgctaatgcctcgtaagag
BhptsG-F	gttctgcaatccagaccttctcttttttttcccgtgacaggtc
BhptsG-R	agagaaggtctggattgcagaacgtgctaatgcctcgtaagag
BhpykA-F	atacgtacaacgttggcacccgcttttttttcccgtgacaggtc
BhpykA-R	gcgggtgccaacgttgtacgtatgtgctaatgcctcgtaagag
BhyjcS-F	cggttctgtcagccacaacgcccttttttttcccgtgacaggtc
BhyjcS-R	gggcgttgtggctgacagaaccggtgctaatgcctcgtaagag
FncadA-F	ccttctacacggccaccgctcatatttcaaataaaacgaaaggctcagtcgaaag
FncadA-R	atgagcggtggccgtgtagaaggatctacaacagtagaaattacatttattgtacaacac
FncynR-F	cccgcccccagttcctgtaacgcatttcaaataaaacgaaaggctcagtcgaaag
FncynR-R	gcgttacaggaactgggggcgggatctacaacagtagaaattacatttattgtacaacac
Fnglk-F	ccgctgtatcgatggcgatcccgatttcaaataaaacgaaaggctcagtcgaaag
Fnglk-R	cgggatcgccatcgatacagcggatctacaacagtagaaattacatttattgtacaacac
FnlacZ-F	cccgctctgctacctgcgccagcatttcaaataaaacgaaaggctcagtcgaaag
FnlacZ-R	gctggcgcaggtagcagagcgggatctacaacagtagaaattacatttattgtacaacac
FnmaeA-F	tgccgcgccgcttcctcgcttaaatttcaaataaaacgaaaggctcagtcgaaag
FnmaeA-R	ttaagcgaggaagcggcgcggcaatctacaacagtagaaattacatttattgtacaacac
FnmhpE-F	gcgtgcttcaccacatccgccgcatttcaaataaaacgaaaggctcagtcgaaag
FnmhpE-R	gcggcggatgtggtgaagcacgcatctacaacagtagaaattacatttattgtacaacac
FnptsG-F	cccgcagtcgggtcacccgccatatttcaaataaaacgaaaggctcagtcgaaag
FnptsG-R	atggcgggtgacccgactgcgggatctacaacagtagaaattacatttattgtacaacac
FnpykA-F	cgcacccaggcaaacgcgcgccaatttcaaataaaacgaaaggctcagtcgaaag

FnpykA-R	tggcgcgcgtttgcctgggtgcgatctacaacagtagaaattacatttattgtacaacac
FnyjcS-F	cccatctccacctgaccatacggatttcaaataaaacgaaaggctcagtcgaaag
FnyjcS-R	ccgtatggtcaggtggagatgggatctacaacagtagaaattacatttattgtacaacac
FnrpsE-F	ccgtaaccaaaaccaacgcgaccatttcaaataaaacgaaaggctcagtcgaaag
FnrpsE-R	ggtcgcgttggttttggttacggatctacaacagtagaaattacatttattgtacaacac
SpcadA-F	caccctgctgactccggggagttttagagctagaaatagcaagttaaaataag
SpcadA-R	tccccggagtcagcagggtgacatttattgtacaacacgagcc
SpcynR-F	tcccaacgtccaactcgtcggttttagagctagaaatagcaagttaaaataag
SpcynR-R	cgacgagttggacgttgggaacatttattgtacaacacgagcc
Spglk-F	gcgccctttatcttcaaatggttttagagctagaaatagcaagttaaaataag
Spglk-R	catttgaagataaagggcgcacatttattgtacaacacgagcc
SplacZ-F	cccgcattgaccctaacgccgttttagagctagaaatagcaagttaaaataag
SplacZ-R	ggcgttagggtcaatgcgggacatttattgtacaacacgagcc
SpmaeA-F	tgcccatcccgccgatgcccgttttagagctagaaatagcaagttaaaataag
SpmaeA-R	gggcatcggcgggatgggcaacatttattgtacaacacgagcc
SpmhpE-F	gcccacggcgacggtttgcagttttagagctagaaatagcaagttaaaataag
SpmhpE-R	tgcaaaccgtcgccgtgggcacatttattgtacaacacgagcc
SpptsG-F	cccagcagaatacctgcgatgttttagagctagaaatagcaagttaaaataag
SpptsG-R	atcgcaggtattctgctgggacatttattgtacaacacgagcc
SppykA-F	cgccctgggtgacaatcaccgttttagagctagaaatagcaagttaaaataag
SppykA-R	ggtgattgtcacccagggcgacatttattgtacaacacgagcc
SprpsE-F	ttccaccaacccgatcaacggttttagagctagaaatagcaagttaaaataag
SprpsE-R	cgttgatcgggttggtggaaacatttattgtacaacacgagcc
FnyjcS-F	cgcgccattgccaataatttgttttagagctagaaatagcaagttaaaataag
FnyjcS-R	aaattattggcaatggcgcgacatttattgtacaacacgagcc
A1-F	ggcagttattgattattgccgtcttttttttcccgtgacaggtc
A1-R	gacggcaataatcaataactgccgtgctaatgcctcgtaagag
A2-F	attattgccgtcatcgttgtactttttttttcccgtgacaggtc
A2-R	agtacaacgatgacggcaataatgtgctaatgcctcgtaagag
A3-F	ccgtcatcgttgtactgctttttttttttcccgtgacaggtc
A3-R	aaaaagcagtacaacgatgacgggtgctaatgcctcgtaagag
A4-F	ctttttaaagcctttgatcgacttttttttcccgtgacaggtc
A4-R	gtcgatcaaaggctttaaaaaaggtgctaatgcctcgtaagag
A5-F	tactgcgaaaactatcgccgatattttttttcccgtgacaggtc
A5-R	tatcggcgatagttttcgcagtagtgctaatgcctcgtaagag
A6-F	gtatccgcctgcttatcggcgattttttttcccgtgacaggtc
A6-R	atcgccgataagcaggcggatacgtgctaatgcctcgtaagag
A7-F	cacctgctctttatcgtggcgcttttttttcccgtgacaggtc
A7-R	agcgccacgataaagagcaggtggtgctaatgcctcgtaagag
A8-F	tatcctgctttggttcatcatcgttttttttcccgtgacaggtc
A8-R	cgatgatgaaccaaagcaggatagtgctaatgcctcgtaagag
A9-F	tagcctgttcctgattcgtatcctttttttcccgtgacaggtc
A9-R	ggatacgaatcaggaacaggctagtgctaatgcctcgtaagag

A10-F	tcgcagtaaaatcagcatcctgatttttttttcccgtgacaggtc
A10-R	tcaggatgctgattttactgcgagtgctaatgcctcgtaagag
B1-F	gtgttcatcatcggcctcgtcgttttttttttcccgtgacaggtc
B1-R	acgacgaggccgatgatgaacacgtgctaatgcctcgtaagag
B2-F	ttaccgccacaggcagtcgttgcttttttttcccgtgacaggtc
B2-R	gcaacgactgcctgtggcggtaagtgctaatgcctcgtaagag
B3-F	tctttcaccaccgggttatggattttttttttcccgtgacaggtc
B3-R	atccataacccggtggtgaaagagtgctaatgcctcgtaagag
B4-F	ctggcttctgttccggcgaactgtttttttttcccgtgacaggtc
B4-R	cagttcgccggaacagaagccaggtgctaatgcctcgtaagag
B5-F	tcggttcagcgtccgcagcaggttttttttttcccgtgacaggtc
B5-R	acctgctgcggacgctgaaccgagtgctaatgcctcgtaagag
C1-F	agctgcgtaagcgtctgctgaactttttttttcccgtgacaggtc
C1-R	gttcagcagacgcttacgcagctgtgctaatgcctcgtaagag
C2-F	ctgtgtctggtctatttcgccaattttttttcccgtgacaggtc
C2-R	ttggcgaaatagaccagacacaggtgctaatgcctcgtaagag
C3-F	cgccaatgacatctatcacctggttttttttcccgtgacaggtc
C3-R	ccaggtgatagatgtcattggcggtgctaatgcctcgtaagag
C4-F	tgtcagcgccggtgattctctattttttttttcccgtgacaggtc
C4-R	atagagaatcaccggcgctgacagtgctaatgcctcgtaagag
C5-F	atcgccccagcgctgtataagcatttttttttcccgtgacaggtc
C5-R	tgcttatacagcgctggggcgatgtgctaatgcctcgtaagag
C6-F	ggcttccttgccaataccgcgcctttttttttcccgtgacaggtc
C6-R	ggcgcggtattggcaaggaagccgtgctaatgcctcgtaagag
C7-F	tcctcttcccgatttcgcccttttttttttttcccgtgacaggtc
C7-R	aaagggcgaaatcgggaagaggagtgctaatgcctcgtaagag
ssTorA-F	catggggggttctatgaacaataacgatctctttcaggc
ssTorA-R	gttcttctcctttgctcgcagtcgcacgtcgcggcg
ssTorA-b-F	cgtgcgactgcgagcaaaggagaagaacttttcactg
ssTorA-b-R	gatcgttattgttcatagaaccccccatggttaattcc

Table S3. The plasmids used in this study

**Plasmids**	**Description**	**References or sources**
pHT01	*E. coli-B. subtilis* shuttle vector, P_{grac} promoter, Cm^R	Lab stock
pHT-BhAIO-sacA	Derived from pHT01, P43-BhCas12b, gRNA targeting *sacA*, Cm^R	This study
pHT-BhAIO-aprE	Derived from pHT01, P43-BhCas12b, gRNA targeting *aprE*, Cm^R	This study
pHT-AaAIO-sacA	Derived from pHT01, P43-AaCas12b, gRNA targeting *sacA*, Cm^R	This study

pAX-dCas9	*B. subtilis* integration vector, derived from pAX01, P_{xylA}-dCas9 expression cassette, Cm^R	Lab stock
pAX-dBhCas12b	Derived from pAX-dCas9, P_{xylA}-dBhCas12b expression cassette, Cm^R	This study
pAX-dBhCas12b-CDA	Derived from pAX-dBhCas12b, P_{xylA}-dBhCas12b-CDA expression cassette, Cm^R	This study
pAX-CDA-dBhCas12b	Derived from pAX-dBhCas12b, P_{xylA}-CDA-dBhCas12b expression cassette, Cm^R	This study
pAX-CDA-dBhCas12b-UGI	Derived from pAX-CDA-dBhCas12b, P_{xylA}-CDA-dBhCas12b-UGI expression cassette, Cm^R	This study
pAX-CDA-dBhCas12b-UGI-UGI	Derived from pAX-CDA-dBhCas12b-UGI, P_{xylA}-CDA-dBhCas12b-UGI-UGI expression cassette, Cm^R	This study
pAX-ABE8e-dBhCas12b	Derived from pAX-dBhCas12b, P_{xylA}-ABE8e-dBhCas12b expression cassette, Cm^R	This study
pHYT	*E. coli*-*B. subtilis* shuttle vector, Tet^R	Lab stock
pHY-pksA	Derived from pHYT, P_{veg}-pksAsgRNA expression cassette, Tet^R	This study
pHY-pksC	Derived from pHYT, P_{veg}-pksCsgRNA expression cassette, Tet^R	This study
pHY-pksE1	Derived from pHYT, P_{veg}-pksE1sgRNA expression cassette, Tet^R	This study
pHY-pksE2	Derived from pHYT, P_{veg}-pksE2sgRNA expression cassette, Tet^R	This study
pHY-pksG1	Derived from pHYT, P_{veg}-pksG1sgRNA expression cassette, Tet^R	This study
pHY-pksG2	Derived from pHYT, P_{veg}-pksG2sgRNA expression cassette, Tet^R	This study
pHY-sigE	Derived from pHYT, P_{veg}-sigEsgRNA expression cassette, Tet^R	This study
pB-P43-eGFP	*E. coli*-*B. subtilis* shuttle vector, P43-eGFP expression cassette, Kan^R	Lab stock
pB-P43-eGFP (K3N)	Derived from pB-P43-eGFP, P43-eGFP (K3N) expression cassette, Kan^R	This study
pB-P43-mRS-eGFP (K3N)	Derived from pB-P43-eGFP (K3N), P43-mRS (G_{15})-eGFP (K3N) expression cassette, Kan^R	This study
pB-P43-mRS-eGFPsg	Derived from pB-P43-mRS-eGFP (K3N), P_{veg}-eGFPsgRNA expression	This study

	cassette, Kan^R	
pB-PylbP-eGFP	*E. coli-B. subtilis* shuttle vector, PylbP-eGFP expression cassette, Kan^R	This study
pB-PrpoB-eGFP	*E. coli-B. subtilis* shuttle vector, PrpoB-eGFP expression cassette, Kan^R	This study
pB-PspoVG-eGFP	*E. coli-B. subtilis* shuttle vector, PspoVG-eGFP expression cassette, Kan^R	This study
pB-PsigW-eGFP	*E. coli-B. subtilis* shuttle vector, PsigW-eGFP expression cassette, Kan^R	This study
pB-PrelA-eGFP	*E. coli-B. subtilis* shuttle vector, PrelA-eGFP expression cassette, Kan^R	This study
pDGT	*B. subtilis* integration vector, P43-eGFP expression cassette, $Spec^R$	Lab stock
pDGT-G1	pDGT derivative, containing gRNA targeting eGFP (G1)	This study
pDGT-G2	pDGT derivative, containing gRNA targeting eGFP (G2)	This study
pDGT-G3	pDGT derivative, containing gRNA targeting eGFP (G3)	This study
pDGT-G4	pDGT derivative, containing gRNA targeting eGFP (G4)	This study
pDGT-G5	pDGT derivative, containing gRNA targeting eGFP (G5)	This study
pDGT-G6	pDGT derivative, containing gRNA targeting eGFP (G6)	This study
pDGT-G7	pDGT derivative, containing gRNA targeting eGFP (G7)	This study
pDGT-G8	pDGT derivative, containing gRNA targeting eGFP (G8)	This study
pDGT-G9	pDGT derivative, containing gRNA targeting eGFP (G9)	This study
pDGT-G10	pDGT derivative, containing gRNA targeting eGFP (G10)	This study
pDGT-G11	pDGT derivative, containing gRNA targeting eGFP (G11)	This study
pDGT-G12	pDGT derivative, containing gRNA targeting eGFP (G12)	This study
pDGT-G13	pDGT derivative, containing gRNA targeting eGFP (G13)	This study
pDGT-G14	pDGT derivative, containing gRNA targeting eGFP (G14)	This study
pDGT-G15	pDGT derivative, containing gRNA targeting eGFP (G15)	This study

pDGT-P43-1	pDGT derivative, containing gRNA targeting P43 promoter (P43-1)	This study
pDGT-P43-2	pDGT derivative, containing gRNA targeting P43 promoter (P43-2)	This study
pDGT-P43-3	pDGT derivative, containing gRNA targeting P43 promoter (P43-3)	This study
pDGT-P43-4	pDGT derivative, containing gRNA targeting P43 promoter (P43-4)	This study
pDGT-ylbP-1	pDGT derivative, containing gRNA targeting PylbP promoter (ylbP-1)	This study
pDGT-ylbP-2	pDGT derivative, containing gRNA targeting PylbP promoter (ylbP-2)	This study
pDGT-ylbP-3	pDGT derivative, containing gRNA targeting PylbP promoter (ylbP-3)	This study
pDGT-ylbP-4	pDGT derivative, containing gRNA targeting PylbP promoter (ylbP-4)	This study
pDGT-spoVG-1	pDGT derivative, containing gRNA targeting PspoVG promoter (spoVG-1)	This study
pDGT-spoVG-2	pDGT derivative, containing gRNA targeting PspoVG promoter (spoVG-2)	This study
pDGT-spoVG-3	pDGT derivative, containing gRNA targeting PspoVG promoter (spoVG-3)	This study
pDGT-spoVG-4	pDGT derivative, containing gRNA targeting PspoVG promoter (spoVG-4)	This study
pDGT-relA-1	pDGT derivative, containing gRNA targeting PrelA promoter (relA-1)	This study
pDGT-relA-2	pDGT derivative, containing gRNA targeting PrelA promoter (relA-2)	This study
pDGT-relA-3	pDGT derivative, containing gRNA targeting PrelA promoter (relA-3)	This study
pDGT-relA-4	pDGT derivative, containing gRNA targeting PrelA promoter (relA-4)	This study
pDGT-rpoB-1	pDGT derivative, containing gRNA targeting PrpoB promoter (rpoB-1)	This study
pDGT-rpoB-2	pDGT derivative, containing gRNA targeting PrpoB promoter (rpoB-2)	This study
pDGT-rpoB-3	pDGT derivative, containing gRNA targeting PrpoB promoter (rpoB-3)	This study
pDGT-sigW-1	pDGT derivative, containing gRNA targeting PsigW promoter (sigW-1)	This study
pDGT-sigW-2	pDGT derivative, containing gRNA targeting PsigW promoter (sigW-2)	This study
pKD46	*E. coli* expression vector, P_{araBAD}-Gam-Beta-Exo expression cassette, Amp^R,	Lab stock

	rep101^{TS}	
pKD-rpsE1	pKD46 derivative, containing P_{araBAD}-CDA-dBhCas12b-UGI expression cassette and sgRNA targeting *rpsE* (E1)	This study
pKD-rpsE2	pKD-rpsE1 derivative, containing P_{araBAD}-CDA-dBhCas12b-UGI expression cassette and sgRNA targeting *rpsE* (E2)	This study
pKD-rpsE3	pKD-rpsE1 derivative, containing P_{araBAD}-CDA-dBhCas12b-UGI expression cassette and sgRNA targeting *rpsE* (E3)	This study
pKD-rpsE4	pKD-rpsE1 derivative, containing P_{araBAD}-CDA-dBhCas12b-UGI expression cassette and sgRNA targeting *rpsE* (E4)	This study
pKD-BhptsG	pKD-rpsE1 derivative, containing P_{araBAD}-CDA-dBhCas12b-UGI expression cassette and sgRNA targeting *ptsG*	This study
pKD-BhcynR	pKD-rpsE1 derivative, containing P_{araBAD}-CDA-dBhCas12b-UGI expression cassette and sgRNA targeting *cynR*	This study
pKD-Bhglk	pKD-rpsE1 derivative, containing P_{araBAD}-CDA-dBhCas12b-UGI expression cassette and sgRNA targeting *glk*	This study
pKD-BhminC	pKD-rpsE1 derivative, containing P_{araBAD}-CDA-dBhCas12b-UGI expression cassette and sgRNA targeting *minC*	This study
pKD-BhlacZ	pKD-rpsE1 derivative, containing P_{araBAD}-CDA-dBhCas12b-UGI expression cassette and sgRNA targeting *lacZ*	This study
pKD-BhcadA	pKD-rpsE1 derivative, containing P_{araBAD}-CDA-dBhCas12b-UGI expression cassette and sgRNA targeting *cadA*	This study
pKD-BhyjcS	pKD-rpsE1 derivative, containing P_{araBAD}-CDA-dBhCas12b-UGI expression cassette and sgRNA	This study

	targeting *yjcS*	
pKD-BhmhpE	pKD-rpsE1 derivative, containing P_{araBAD}-CDA-dBhCas12b-UGI expression cassette and sgRNA targeting *mhpE*	This study
pKD-BhpykA	pKD-rpsE1 derivative, containing P_{araBAD}-CDA-dBhCas12b-UGI expression cassette and sgRNA targeting *pykA*	This study
pKD-BhmaeA	pKD-rpsE1 derivative, containing P_{araBAD}-CDA-dBhCas12b-UGI expression cassette and sgRNA targeting *maeA*	This study
pKD-FnptsG	pKD46 derivative, containing P_{araBAD}-CDA-dFnCas12a-UGI expression cassette and crRNA targeting *ptsG*	This study
pKD-FncynR	pKD-FnptsG derivative, containing P_{araBAD}-CDA-dFnCas12a-UGI expression cassette and crRNA targeting *cynR*	This study
pKD-Fnglk	pKD-FnptsG derivative, containing P_{araBAD}-CDA-dFnCas12a-UGI expression cassette and crRNA targeting *glk*	This study
pKD-FnrpsE	pKD-FnptsG derivative, containing P_{araBAD}-CDA-dFnCas12a-UGI expression cassette and crRNA targeting *rpsE*	This study
pKD-FnlacZ	pKD-FnptsG derivative, containing P_{araBAD}-CDA-dFnCas12a-UGI expression cassette and crRNA targeting *lacZ*	This study
pKD-FncadA	pKD-FnptsG derivative, containing P_{araBAD}-CDA-dFnCas12a-UGI expression cassette and crRNA targeting *cadA*	This study
pKD-FnyjcS	pKD-FnptsG derivative, containing P_{araBAD}-CDA-dFnCas12a-UGI expression cassette and crRNA targeting *yjcS*	This study
pKD-FnmhpE	pKD-FnptsG derivative, containing P_{araBAD}-CDA-dFnCas12a-UGI expression cassette and crRNA targeting *mhpE*	This study

pKD-FnpykA	pKD-FnptsG derivative, containing P_{araBAD}-CDA-dFnCas12a-UGI expression cassette and crRNA targeting *pykA*	This study
pKD-FnmaeA	pKD-FnptsG derivative, containing P_{araBAD}-CDA-dFnCas12a-UGI expression cassette and crRNA targeting *maeA*	This study
pKD-SpptsG	pKD46 derivative, containing P_{araBAD}-CDA-dSpCas9-UGI expression cassette and sgRNA targeting *ptsG*	This study
pKD-SpcynR	pKD-SpptsG derivative, containing P_{araBAD}-CDA-dSpCas9-UGI expression cassette and sgRNA targeting *cynR*	This study
pKD-Spglk	pKD-SpptsG derivative, containing P_{araBAD}-CDA-dSpCas9-UGI expression cassette and sgRNA targeting *glk*	This study
pKD-SprpsE	pKD-SpptsG derivative, containing P_{araBAD}-CDA-dSpCas9-UGI expression cassette and sgRNA targeting *rpsE*	This study
pKD-SplacZ	pKD-SpptsG derivative, containing P_{araBAD}-CDA-dSpCas9-UGI expression cassette and sgRNA targeting *lacZ*	This study
pKD-SpcadA	pKD-SpptsG derivative, containing P_{araBAD}-CDA-dSpCas9-UGI expression cassette and sgRNA targeting *cadA*	This study
pKD-SpyjcS	pKD-SpptsG derivative, containing P_{araBAD}-CDA-dSpCas9-UGI expression cassette and sgRNA targeting *yjcS*	This study
pKD-SpmhpE	pKD-SpptsG derivative, containing P_{araBAD}-CDA-dSpCas9-UGI expression cassette and sgRNA targeting *mhpE*	This study
pKD-SppykA	pKD-SpptsG derivative, containing P_{araBAD}-CDA-dSpCas9-UGI expression cassette and sgRNA targeting *pykA*	This study
pKD-SpmaeA	pKD-SpptsG derivative, containing P_{araBAD}-CDA-dSpCas9-UGI expression cassette and sgRNA targeting *maeA*	This study
pKD-BhtatA-1	pKD-rpsE1 derivative, containing P_{araBAD}-CDA-dBhCas12b-UGI expression cassette and sgRNA targeting *tatA-1*	This study
pKD-BhtatA-2	pKD-rpsE1 derivative, containing P_{araBAD}-CDA-dBhCas12b-UGI	This study

	expression cassette and sgRNA targeting *tatA-2*	
pKD-BhtatA-3	pKD-rpsE1 derivative, containing P_{araBAD}-CDA-dBhCas12b-UGI expression cassette and sgRNA targeting *tatA-3*	This study
pKD-BhtatA-4	pKD-rpsE1 derivative, containing P_{araBAD}-CDA-dBhCas12b-UGI expression cassette and sgRNA targeting *tatA-4*	This study
pKD-BhtatA-5	pKD-rpsE1 derivative, containing P_{araBAD}-CDA-dBhCas12b-UGI expression cassette and sgRNA targeting *tatA-5*	This study
pKD-BhtatA-6	pKD-rpsE1 derivative, containing P_{araBAD}-CDA-dBhCas12b-UGI expression cassette and sgRNA targeting *tatA-6*	This study
pKD-BhtatA-7	pKD-rpsE1 derivative, containing P_{araBAD}-CDA-dBhCas12b-UGI expression cassette and sgRNA targeting *tatA-7*	This study
pKD-BhtatA-8	pKD-rpsE1 derivative, containing P_{araBAD}-CDA-dBhCas12b-UGI expression cassette and sgRNA targeting *tatA-8*	This study
pKD-BhtatA-9	pKD-rpsE1 derivative, containing P_{araBAD}-CDA-dBhCas12b-UGI expression cassette and sgRNA targeting *tatA-9*	This study
pKD-BhtatA-10	pKD-rpsE1 derivative, containing P_{araBAD}-CDA-dBhCas12b-UGI expression cassette and sgRNA targeting *tatA-10*	This study
pKD-BhtatB-1	pKD-rpsE1 derivative, containing P_{araBAD}-CDA-dBhCas12b-UGI expression cassette and sgRNA targeting *tatB-1*	This study
pKD-BhtatB-2	pKD-rpsE1 derivative, containing P_{araBAD}-CDA-dBhCas12b-UGI expression cassette and sgRNA targeting *tatB-2*	This study
pKD-BhtatB-3	pKD-rpsE1 derivative, containing P_{araBAD}-CDA-dBhCas12b-UGI	This study

	expression cassette and sgRNA targeting *tatB-3*	
pKD-BhtatB-4	pKD-rpsE1 derivative, containing P_{araBAD}-CDA-dBhCas12b-UGI expression cassette and sgRNA targeting *tatB-4*	This study
pKD-BhtatB-5	pKD-rpsE1 derivative, containing P_{araBAD}-CDA-dBhCas12b-UGI expression cassette and sgRNA targeting *tatB-5*	This study
pKD-BhtatC-1	pKD-rpsE1 derivative, containing P_{araBAD}-CDA-dBhCas12b-UGI expression cassette and sgRNA targeting *tatC-1*	This study
pKD-BhtatC-2	pKD-rpsE1 derivative, containing P_{araBAD}-CDA-dBhCas12b-UGI expression cassette and sgRNA targeting *tatC-2*	This study
pKD-BhtatC-3	pKD-rpsE1 derivative, containing P_{araBAD}-CDA-dBhCas12b-UGI expression cassette and sgRNA targeting *tatC-3*	This study
pKD-BhtatC-4	pKD-rpsE1 derivative, containing P_{araBAD}-CDA-dBhCas12b-UGI expression cassette and sgRNA targeting *tatC-4*	This study
pKD-BhtatC-5	pKD-rpsE1 derivative, containing P_{araBAD}-CDA-dBhCas12b-UGI expression cassette and sgRNA targeting *tatC-5*	This study
pKD-BhtatC-6	pKD-rpsE1 derivative, containing P_{araBAD}-CDA-dBhCas12b-UGI expression cassette and sgRNA targeting *tatC-6*	This study
pKD-BhtatC-7	pKD-rpsE1 derivative, containing P_{araBAD}-CDA-dBhCas12b-UGI expression cassette and sgRNA targeting *tatC-7*	This study
pBAD-ssTorA-sfGFP	*E. coli* expression vector, P_{araBAD}-ssTorA-sfGFP expression cassette, Amp^R	This study

Table S4. The promoter sequences used in this study

Promoter	Sequences (5'-3')
P43	AGCTGTAATGGCTGAAAATTCTTACATTTATTTTACATTTTTAGAAATGGGCGTGAAAAAAAGCGCGCGATTATGTAAAATATAAAGTGATAGCGGTAC
PylbP	TAAAGTTTAAATATTTGGATTTTTTAAATAAAGCGTTTACAATATATGTAGAAACAACAA
PrpoB	AAGCAAAAAAAGTTTGACTCGGTATTTTAACTATGTTAATATTGTAAAATGCCAATGTAT
PrelA	TCCTATTGTTTTGCATTTATTTTATATAATATTTGGCTATTTGAACTTCTGCTCTTTACA
PspoVG	ATTTTAAAAACGAGCAGGATTTCAGAAAAAATCGTGGAATTGATACACTAATGCTTTTAT
PsigW	ATTTTATAAAAAAATTGAAACCTTTTGAAACGAAGCTCGTATACATACAGACCGGTGAAG

Annotations:

The underlines indicate the core region of the target promoter.

Table S5. The protospacer sequences used in this study

sgRNA sequences (5'-3')	PAM	Purpose
CCACATGTATCCAAAATCGTCCA	ATTC	Targeting *sacA* (BhCas12b)
TACAGCGCTGAAAACAGTCGTTG	GTTC	Targeting *aprE* (BhCas12b)
ACCATGCTTCAGTTCAGGCT	ATTC	Targeting *sacA* (AaCas12b)
TTCTCCCTTACCCATTTTTTTTC	GTTC	Targeting eGFP (G1)
AACAAGAATTGGGACAACTCCAG	ATTC	Targeting eGFP (G2)
TTGTTGAATTAGATGGTGATGTT	ATTC	Targeting eGFP (G3)
ATGGGCACAAATTTTCTGTCAGT	GTTA	Targeting eGFP (G4)
TCTGTCAGTGGAGAGGGTGAAGG	ATTT	Targeting eGFP (G5)
GCCAAGGAACAGGCAGCTTTCCA	GTTG	Targeting eGFP (G6)
CTTGGCCAACACTTGTCACTACT	GTTC	Targeting eGFP (G7)
CCTTAAGCTCGATTCTGTTGACG	ATTC	Targeting eGFP (G8)
CAAGGAGGACGGAAACATCCTCG	ATTT	Targeting eGFP (G9)
TGTCTAATTTTGAAGTTAACTTT	GTTG	Targeting eGFP (G10)
ACTTCAAAATTAGACACAACATT	GTTA	Targeting eGFP (G11)
TGTTGATAATGGTCTGCTAGTTG	ATTT	Targeting eGFP (G12)
TCAACAAAATACTCCAATTGGCG	ATTA	Targeting eGFP (G13)
TGTGGACAGGTAATGGTTGTCTG	ATTG	Targeting eGFP (G14)
CCTGTCCACACAATCTGCCCTTT	ATTA	Targeting eGFP (G15)
CTAAAAATGTAAAATAAATGTAA	ATTT	Targeting P43 promoter (P43-1)

TTAGAAATGGGCGTGAAAAAAAG	ATTT	Targeting P43 promoter (P43-2)
TACATAATCGCGCGCTTTTTTTC	ATTT	Targeting P43 promoter (P43-3)
TGTAAAATATAAAGTGATAGCGG	ATTA	Targeting P43 promoter (P43-4)
AAAAAATCCAAATATTTAAACTT	ATTT	Targeting PylbP promoter (ylbP-1)
GGATTTTTTAAATAAAGCGTTTA	ATTT	Targeting PylbP promoter (ylbP-2)
TTTAAATAAAGCGTTTACAATAT	ATTT	Targeting PylbP promoter (ylbP-3)
ACAATATATGTAGAAACAACAAC	GTTT	Targeting PylbP promoter (ylbP-4)
TTTTGCATTTATTTTATATAATA	ATTG	Targeting PrelA promoter (relA-1)
TATAAAATAAATGCAAAACAATA	ATTA	Targeting PrelA promoter (relA-2)
GAACTTCTGCTCTTTACACCTCG	ATTT	Targeting PrelA promoter (relA-3)
GGCTATTTGAACTTCTGCTCTTT	ATTT	Targeting PrelA promoter (relA-4)
TAAAAACGAGCAGGATTTCAGAA	ATTT	Targeting PspoVG promoter (spoVG-1)
CACGATTTTTTCTGAAATCCTGC	ATTC	Targeting PspoVG promoter (spoVG-2)
CAGAAAAAATCGTGGAATTGATA	ATTT	Targeting PspoVG promoter (spoVG-3)
ATACACTAATGCTTTTATcCTCG	ATTG	Targeting PspoVG promoter (spoVG-4)
ACATAGTTAAAATACCGAGTCAA	ATTA	Targeting PrpoB promoter (rpoB-1)
TAACTATGTTAATATTGTAAAAT	ATTT	Targeting PrpoB promoter (rpoB-2)
GCATTTTACAATATTAACATAGT	ATTG	Targeting PrpoB promoter (rpoB-3)
TATAAAAAAATTGAAACCTTTTG	ATTT	Targeting PsigW promoter (sigW-1)
AAACCTTTTGAAACGAAGCTCGT	ATTG	Targeting PsigW promoter (sigW-2)
CTGGCTGATGCTCCCTCCATCCC	ATTT	Targeting *pksA* (BhCas12b)
CTTATCAGACTCATCAGCTCGCC	GTTG	Targeting *pksC* (BhCas12b)
AAATAGCCCTTGTCCCATCCCCT	GTTC	Targeting *pksE1*

		(BhCas12b)
CGCCCCCTGTAATTCGTCCCATC	ATTC	Targeting *pksE2* (BhCas12b)
TCGCCAACTAAAACGGCCACCGC	ATTT	Targeting *pksG1* (BhCas12b)
CAATATCTTGCTTACCATACGCC	GTTT	Targeting *pksG2* (BhCas12b)
TCTAAAGATGAGGAGCAGGTTTT	ATTA	Targeting *sigE* (E1)
AATCCAGAAAAGAAAATCAAGCT	ATTT	Targeting *sigE* (E2)
AAGAAGAAATAACAAAATCCGTT	ATTT	Targeting *sigE* (E3)
CCCCAGAATTTCTTCAACGGATT	ATTT	Targeting *rpsE* (E1)
CACCAACCCGATCAACGTGGTTC	GTTC	Targeting *rpsE* (E2)
TCTCCTTCACAGCTCTGACTGTT	ATTT	Targeting *rpsE* (E3)
TAGATACGCGGTTTACCGCGATC	GTTT	Targeting *rpsE* (E4)
CACCCTGCTGACTCCGGGGA	TGG	Targeting *cadA* (SpCas9)
TCCCAACGTCCAACTCGTCG	CGG	Targeting *cynR* (SpCas9)
GCGCCCTTTATCTTCAAATG	CGG	Targeting *glk* (SpCas9)
CCCGCATTGACCCTAACGCC	TGG	Targeting *lacZ* (SpCas9)
TGCCCATCCCGCCGATGCCC	TGG	Targeting *maeA* (SpCas9)
GCCCACGGCGACGGTTTGCA	GGG	Targeting *mhpE* (SpCas9)
CCCAGCAGAATACCTGCGAT	AGG	Targeting *ptsG* (SpCas9)
CGCCCTGGGTGACAATCACC	AGG	Targeting *pykA* (SpCas9)
TTCCACCAACCCGATCAACG	TGG	Targeting *rpsE* (SpCas9)
CGCGCCATTGCCAATAATTT	TGG	Targeting *yjcS* (SpCas9)
CCTTCTACACGGCCACCGCTCAT	TTTC	Targeting *cadA* (FnCas12a)
CCCGCCCCCAGTTCCTGTAACGC	TTTA	Targeting *cynR* (FnCas12a)
CCGCTGTATCGATGGCGATCCCG	TTTA	Targeting *glk* (FnCas12a)
CCCGCTCTGCTACCTGCGCCAGC	TTTA	Targeting *lacZ* (FnCas12a)
TGCCGCGCCGCTTCCTCGCTTAA	TTTC	Targeting *maeA* (FnCas12a)
GCGTGCTTCACCACATCCGCCGC	TTTG	Targeting *mhpE* (FnCas12a)
CCCGCAGTCGGGTCACCCGCCAT	TTTA	Targeting *ptsG* (FnCas12a)
CGCACCCAGGCAAACGCGCGCCA	TTTC	Targeting *pykA* (FnCas12a)
CCGTAACCAAAACCAACGCGACC	TTTA	Targeting *rpsE* (FnCas12a)
CCCATCTCCACCTGACCATACGG	TTTA	Targeting *yjcS* (FnCas12a)
CACCCGGCATTACCAGAGGAACT	ATTT	Targeting *cadA* (BhCas12b)
CCACACAACATGACGGGCTTAAA	ATTG	Targeting *cynR* (BhCas12b)
GTCATCGCCACCCAGTCACCGGT	GTTG	Targeting *glk* (BhCas12b)
CAGCTGAGCGCCGGTCGCTACCA	ATTC	Targeting *lacZ* (BhCas12b)
AACGGGCTGCCCGTGGCGACCAG	ATTA	Targeting *maeA* (BhCas12b)
CCGCCCAGCATATTCAGTATGCC	GTTT	Targeting *mhpE* (BhCas12b)
TCCATCAGGCGCTGGAAGACAAA	GTTA	Targeting *minC* (BhCas12b)
GTTCTGCAATCCAGACCTTCTCT	ATTG	Targeting *ptsG* (BhCas12b)

ATACGTACAACGTTGGCACCCGC	GTTC	Targeting *pykA* (BhCas12b)
CGGTTCTGTCAGCCACAACGCCC	ATTA	Targeting *yjcS* (BhCas12b)
GGCAGTTATTGATTATTGCCGTC	ATTT	Targeting *tatA* (A1)
ATTATTGCCGTCATCGTTGTACT	ATTG	Targeting *tatA* (A2)
CCGTCATCGTTGTACTGCTTTTT	ATTG	Targeting *tatA* (A3)
CTTTTTTAAAGCCTTTGATCGAC	ATTG	Targeting *tatA* (A4)
TACTGCGAAAACTATCGCCGATA	ATTT	Targeting *tatA* (A5)
GTATCCGCCTGCTTATCGGCGAT	ATTC	Targeting *tatA* (A6)
CACCTGCTCTTTATCGTGGCGCT	ATTA	Targeting *tatA* (A7)
TATCCTGCTTTGGTTCATCATCG	GTTT	Targeting *tatA* (A8)
TAGCCTGTTCCTGATTCGTATCC	GTTT	Targeting *tatA* (A9)
TCGCAGTAAAATCAGCATCCTGA	GTTT	Targeting *tatA* (A10)
GTGTTCATCATCGGCCTCGTCGT	ATTG	Targeting *tatB* (B1)
TTACCGCCACAGGCAGTCGTTGC	GTTT	Targeting *tatB* (B2)
TCTTTCACCACCGGGTTATGGAT	ATTA	Targeting *tatB* (B3)
CTGGCTTCTGTTCCGGCGAACTG	GTTT	Targeting *tatB* (B4)
TCGGTTCAGCGTCCGCAGCAGGT	GTTT	Targeting *tatB* (B5)
AGCTGCGTAAGCGTCTGCTGAAC	ATTG	Targeting *tatC* (C1)
CTGTGTCTGGTCTATTTCGCCAA	ATTC	Targeting *tatC* (C2)
CGCCAATGACATCTATCACCTGG	ATTT	Targeting *tatC* (C3)
TGTCAGCGCCGGTGATTCTCTAT	ATTC	Targeting *tatC* (C4)
ATCGCCCCAGCGCTGTATAAGCA	ATTT	Targeting *tatC* (C5)
GGCTTCCTTGCCAATACCGCGCC	ATTT	Targeting *tatC* (C6)
TCCTCTTCCCGATTTCGCCCTTT	GTTT	Targeting *tatC* (C7)
CCCATCCCCCCCCCCCCCCCTCT	ATTA	Targeting RS of eGFP

Table S6. The off-target analysis of dSpCas9-CBE, dFnCas12a-CBE, and dBhCas12b-CBE in *E. coli*

	Loci & target sequence (5'-3')	Similar DNA sequence[a]	Positions[b]	Mismatches[c]	Positive[d]
dSpCas9-CBE	*cynR* TCCCAACGTCCAACTCGTCGCGG	TaCCgACaTCCAgacCGTCG	34482	6	N. D.
		TgCCgAtaTCCAACTCtTCc	522624	6	N. D.
		TCCttACGTCaAACTgcTgG	949971	6	N. D.
		cCCCgtCGTCgAAtcCGT	1187985	6	N. D.

		CG			
		TCCCggCGaCCAtCgCGTCa	1198411	6	N. D.
		TCagcACGcCCAAacCGTCG	1499346	6	N. D.
		TaCCAACaTaCAgggCGTCG	1657827	6	N. D.
		cgCCAACGTCCAgCgCGcCa	2064258	6	N. D.
		TCtCAACGTCCAtaTgaTgG	2207114	6	N. D.
		ggCCAACGgCgAAgTCGgCG	2392533	6	N. D.
		aCCaAcCGTCCAgCcaGTCG	3192666	6	N. D.
		cCCCAtCGcCaAACTtGTCc	4397130	6	N. D.
		aCCCAACaTgCAgCgCGTaG	4388044	6	N. D.
		cCgCcAgGcCCAAaTCGTCG	4558731	6	N. D.
dSpCas9-CBE	*maeA* TGCCCATCCCGCCGATGCCCTGG	TGCCgATCtgatCGATGCCC	162900	5	N. D.
		TGgCgATCCCGCCGtTGtaC	228373	5	N. D.
		TGCCgtTCCCGCCagTGCtC	485388	5	N. D.
		TcCCaATaCCGCCGATcgCC	716162	5	N. D.
		TGCCCATC	2462429	5	N. D.

		aCaCgaATGCgC			
		gGCggATgCCGCCGATGCgC	2694686	5	N. D.
		TGCCCgTatCGtCGATGCgC	2685931	5	N. D.
		gGgtgATCCCGCCtATGCCC	2864090	5	N. D.
		cGCCCgcCtCGCCGATGaC	3099735	5	N. D.
		TGtCCATCtCGaCaAaGCCC	3664459	5	N. D.
		TGCCCcTgtCGCCGATGCga	4373583	5	N. D.
		TGCgCATCCCGCCtgcGCCC	3833036	4	N. D.
		TGCCgATaCCGCCGtaGCgC	4128275	5	N. D.
dSpCas9-CBE	*yjcS* CGCGCCATTGCCAATAATTTTGG	CcCGaaATcGCCAtTAATTT	1564559	5	N. D.
		CGCagtATTGCCAATAATga	2370348	5	N. D.
		CGCGCCATTGCCcATctTgg	2835125	5	N. D.
		CGCGCCAgaGCaAATAAcTg	4563646	5	N. D.
dFnCas12a-CBE	*cynR* TTTACCCGCCCCCAGTTCCTGTAACGC	CgCGCCgCCAGTTtCgGcAACGC	2138625	5	N. D.
		CCCagCgCCtGcTCCaGTAACGC	3836	6	N. D.

		CCCGttCCC gGTTgCTGT AcCGg	1487399	6	N. D.
		CCCGCaCC CAGTTCgg GTcAacC	1688549	6	N. D.
		CCtcaCCgC AGaTCCTG cAACGC	4315186	6	N. D.
		CCCGCtaCC AGcgCagcT AACGC	271330	7	N. D.
		CtCGCCCag cGgcCCTGT AACcC	1370072	7	N. D.
		aCgtCCgCC AGTTCCgG cAACGt	2481186	7	N. D.
		tttGCCaCCt GcTCCTGc AACGC	2446700	7	N. D.
		tCCGgCgtCt GTTggTGTA ACGC	2579879	7	N. D.
		CCaGaggCC AGTTCCgG agACGC	3499781	7	N. D.
		CCCGgCtCC tGgTCggGT AACtC	3979286	7	N. D.
dFnCas12a-CBE	*maeA* TTTCTGCC GCGCCGCT TCCTCGCT TAA	TGCCGCGC tGCTgCacat CTTAA	2033700	6	N. D.
		TGCaGCGC CGCTTtCTC GtaagA	2238971	6	N. D.
		TGgCGCagC GaTTgCTCG CcgAA	475522	7	N. D.
		aaCaGCGtC GtTTCCagG CTTAA	861629	7	N. D.
		aGCCttGCC GCTTtaTgG	1185872	7	N. D.

		CgTAA			
		TGCCGCGt CtaTTCtTtG CcgAA	1301284	7	N. D.
		TctgGCGCg GCaTCCTg GCcTAA	1587091	7	N. D.
		gGgCGCGC CGCTgCCcg cCTgAA	2972117	7	N. D.
		gGCaatGCC GCTTtCTCG CTTgc	3530722	7	N. D.
		TaCCGCGC CGCcTgCTa cCggAA	3957421	7	N. D.
dFnCas12a-CBE	*yjcS* TTTACCCATCTCCACCTGACCATACGG	tCgATCTCC ACtTcAgCA TACGt	2220023	6	N. D.
		CtCATCTtCc aCTaACCAg ACcG	5607	7	N. D.
		aCCATgTgC ACCgGttCcT ACGG	89190	7	N. D.
		tCCATCTtCg CCTGAgCA gAaGa	1164563	7	N. D.
		aCCATaTCa ACaTGACC AaAgcG	1995305	7	N. D.
		aCCATCTCa tCgTaACCA TcCtG	3626260	7	N. D.
dBhCas12b-CBE	*cynR* ATTGCCACACAACATGACGGGCTTAAA	CCgCAtcAC ATaACGGG CTTgct	522096	7	N. D.
		CCACAaAtC ATtAgcGGtT TtAA	571933	7	N. D.
		tCAaAaAAa ATGACGGG gaTAAc	1374385	7	N. D.
		CCACgCAc	2788084	7	N. D.

		CgTcgCGGGgaTAAA			
		ggACtCAcCATGgCGGGaTgAAA	3286678	7	N. D.
		tCACtggcaATGACGGGCTTAAc	4022401	7	N. D.
dBhCas12b-CBE	*maeA* ATTAAACGGGCTGCCCGTGGCGACCAG	AACtGGCgGCCCGcGcaGAaCAG	183256	6	N. D.
		ctgcGGCTGaCCGTGGCttCCAG	343648	7	N. D.
		AcCGtGCTGCtgtTGGCGgtCAG	809819	7	N. D.
		AAgGtGCTGaCCtTaGCGcCaAG	1146676	7	N. D.
		cACacGCTGCCacTGGCaACCAa	1366051	7	N. D.
		AAgtcGtTGaCCaTGGtGACCAG	1399298	7	N. D.
		AAaGGtaTGgCgcTGGCGACCAt	2022655	7	N. D.
		AAtaGGgTGCCgGTGcCaAtCAG	2137320	7	N. D.
		AcCGctCTGCCgGTGGttACCAt	4281518	7	N. D.
dBhCas12b-CBE	*yjcS* ATTACGGTTCTGTCAGCCACAACGCCC	CGtTgCcGcCtGCCACAACGCCa	3604459	6	N. D.
		tctTTCgGTCAGCtAaAAtGCCC	812734	7	N. D.
		CGGTcCaGTCAaCaAaAACaCgC	1173490	7	N. D.

		tGcTTCTGT CAcatcCcAC GCCC	2009226	7	N. D.
		CGGTTaTG TaAcCCgCt AtGaCC	3910410	7	N. D.

Annotations:

a: Choromosomal areas most similar to target sequence were predicted by Cas-OFFinder

b: Genome positions of *Bacillus subtilis* 168 predicted by Cas-OFFinder

c: The number of mismatched bases between target region and off-target region

d: The designated off-target Positions were detected by Next-Generation Sequencing (NGS)

N.D.: Not detection

The PAM motifs were indicated in red.

Supplementary Figures

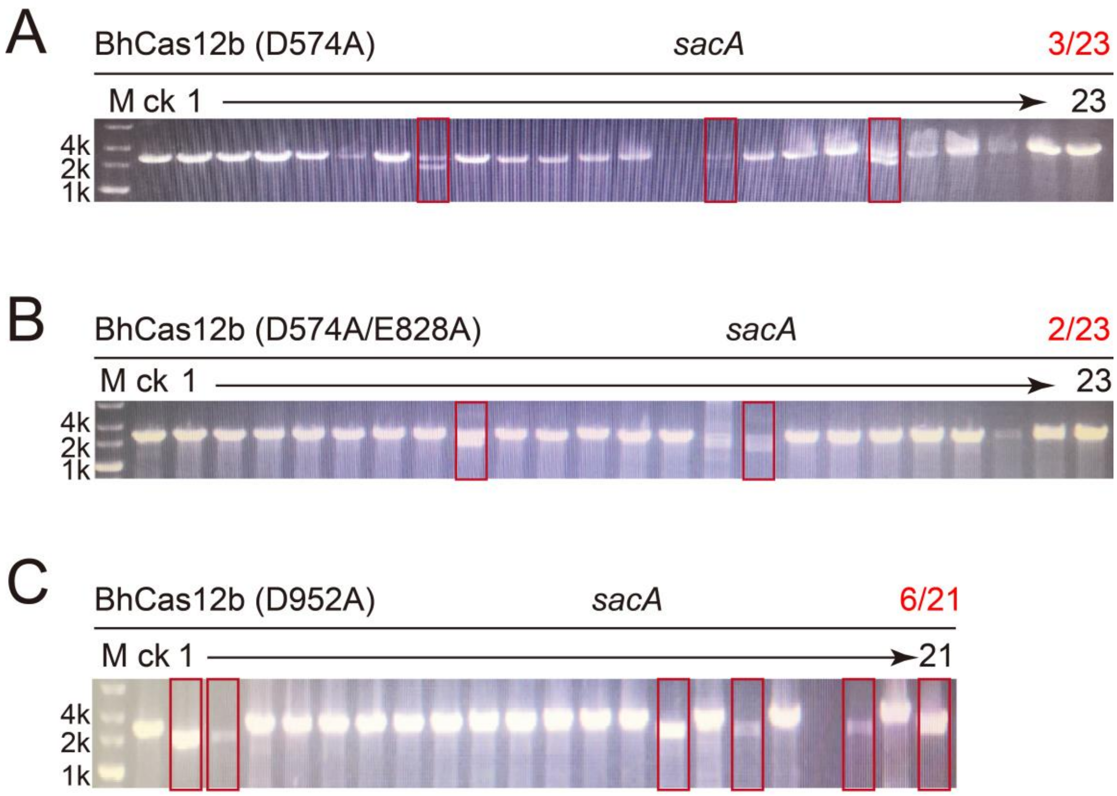

Figure S1 Investigation of the *sacA* deletion efficiency for different variants of

BhCas12b.

Comparison of *sacA* deletion efficiency between BhCas12b (D574A) (**A**), BhCas12b (D574A/E828A) (**B**), and BhCas12b (D952A) (**C**).

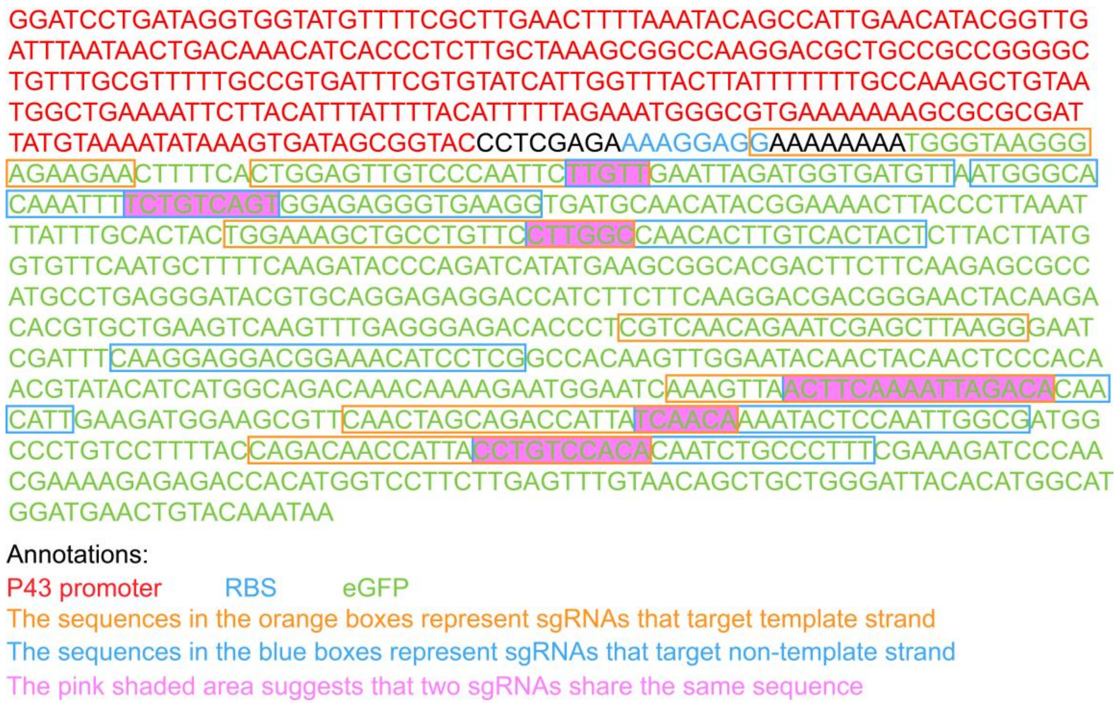

Figure S2 The relevant elements of eGFP expression cassette and the positions of sgRNA in the eGFP coding region.

Relevant elements are labeled in different colors.

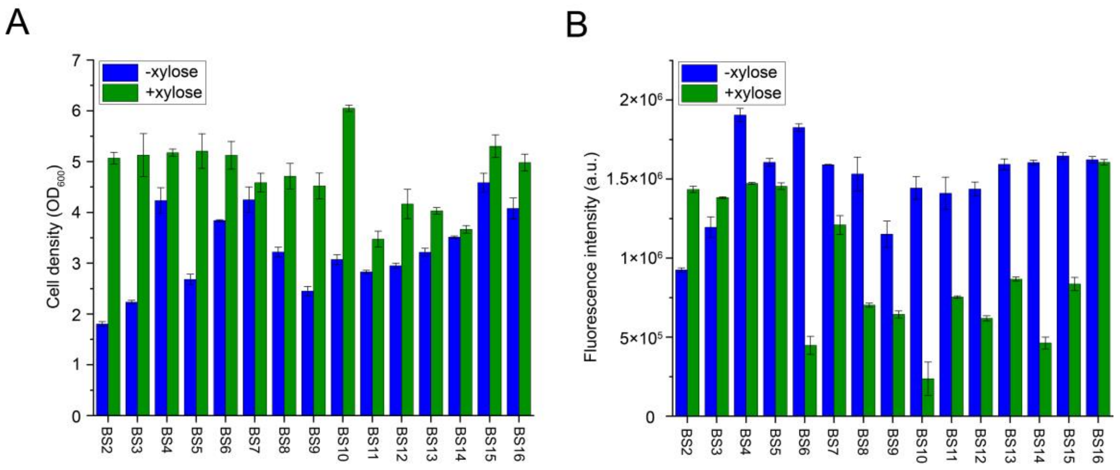

Figure S3 Analysis of cell density and total fluorescence intensity using dBhCas12b-based CRISPRi system.

A: Cell density of the recombinant strains with or without 1% inducer xylose. **B:** Total

FI of the recombinant strains with or without 1% inducer xylose.

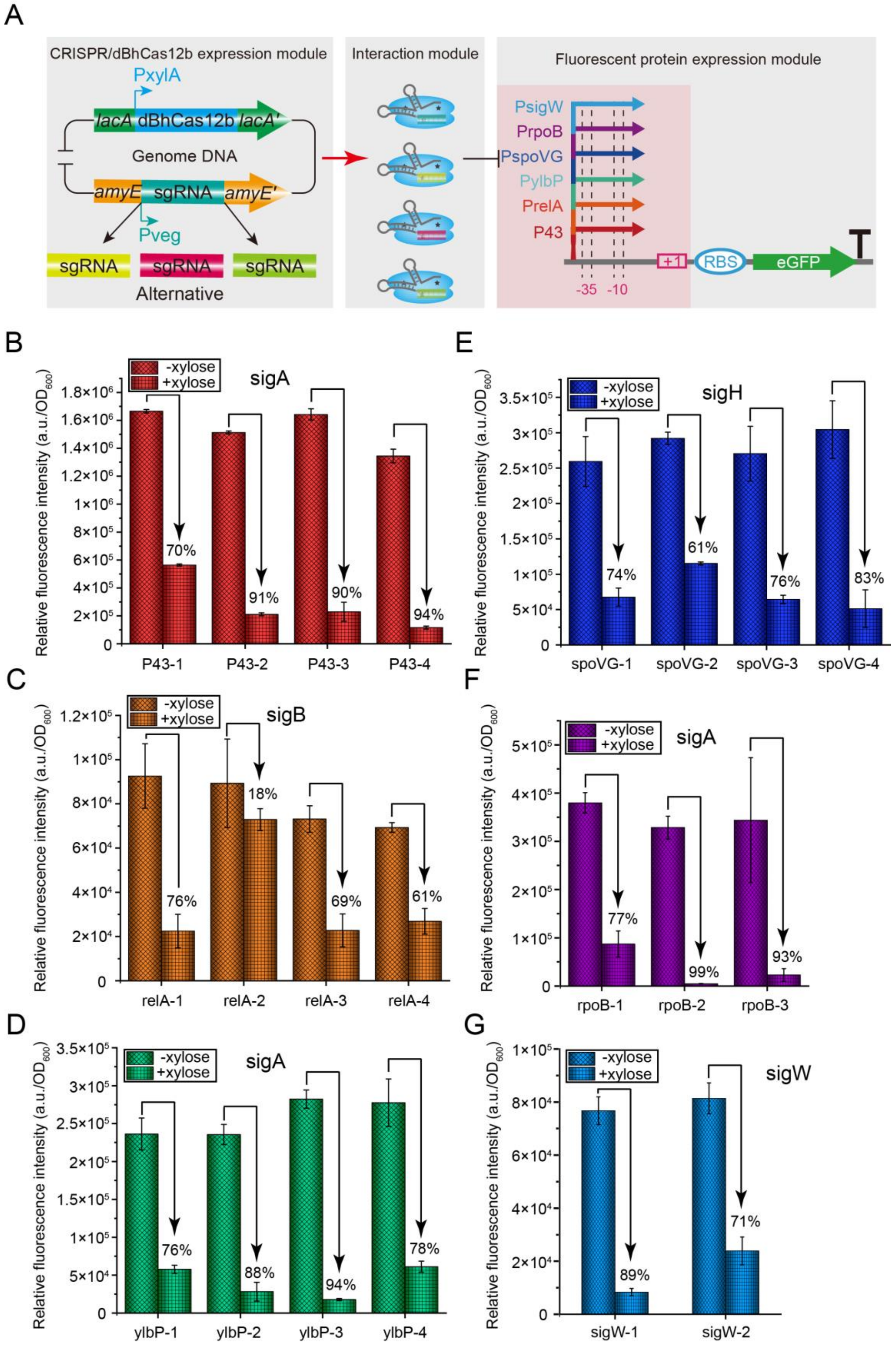

Figure S4 Analysis of transcription initiation repression by dBhCas12b-based

CRISPRi system

A: Three modules (expression of CRISPRi, interaction and fluorescent protein expression) of the repression of transcription initiation. **B-G:** RFI of the recombinant strains targeting different promoters. Six endogenous promoters (P43, Pylbp, PrpoB, PrelA, PspoVG, and PsigW) were selected as targets. 21 sgRNAs targeting the core regions of these 6 promoters were designed and integrated them into the *amyE* locus of BS1, resulting in 21 recombinant strains (BS17 to BS37). Six fluorescence-based reporter plasmids, PP43-eGFP, PrelA-eGFP, PylbP-eGFP, PspoVG-eGFP, PrpoB-eGFP, and PsigW-eGFP, were transformed into the corresponding recombinant strains, and the RFI was determined.

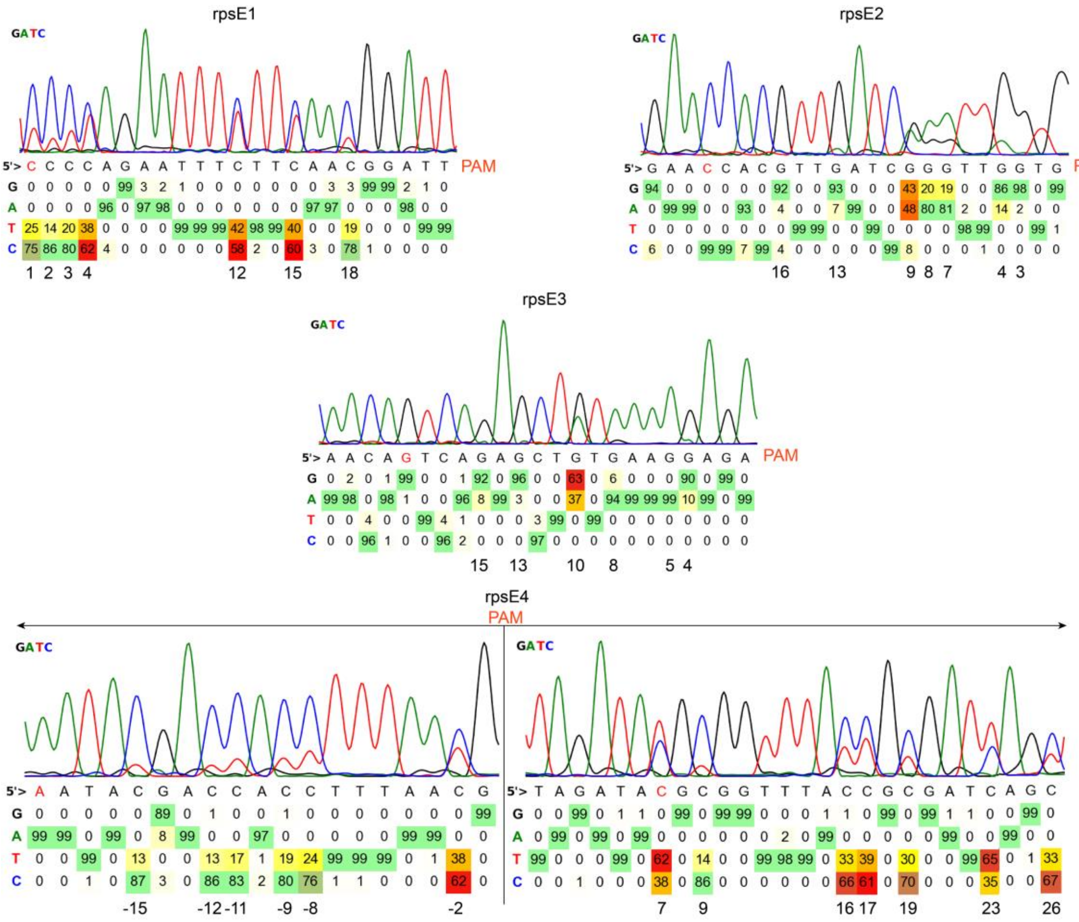

Figure S5 Sample image output of the sequencing data for the editing of *rpsE* from the BEAT analysis

Editing efficiencies of the dBhCas12b-based CBE for targeting different sites of *rpsE*. The raw sequencing data were quantitatively analyzed by BEAT[1]. The mutated bases are numbered, and the positions of the PAM motifs are displayed in red.

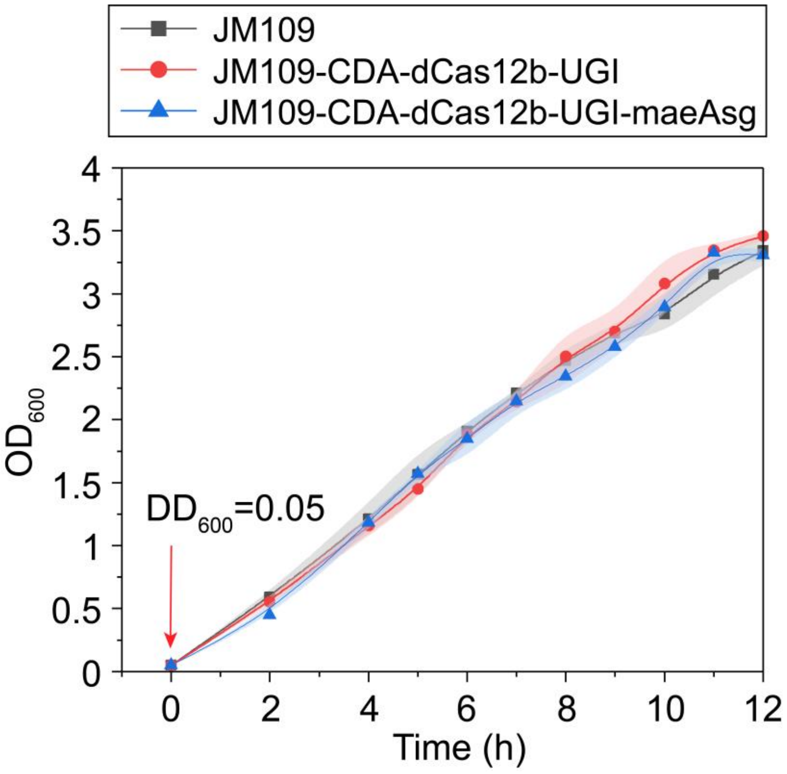

Figure S6 The impact of dBhCas12b on the growth of *E. coli*.

All strains are induced by arabinose when the OD_{600} is 0.05. Data are shown as the mean± s.d. from three independent experiments.

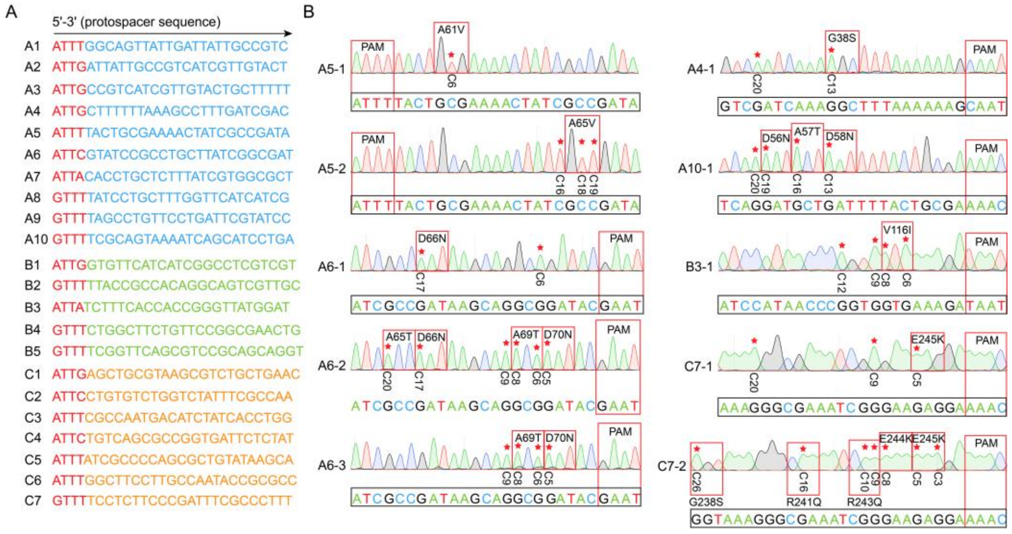

Figure S7 Sequencing results for the Tat translocase mutants isolated from the mutant library

A: All sgRNA sequences targeting TatABC. **B:** Identification of mutants by sequencing. The mutated bases are numbered and indicated in red stars. The corresponding amino acid replacements are framed with red rectangles.

Supplementary Methods

Plasmid construction for gene deletion in *B. subtilis* (CRISPR-BhCas12b):

The bacterial codon-optimized BhCas12b was amplified using the primers BhCas12b-F and BhCas12b-R, while the backbone of pHTsacA, harboring the *sacA* homologous arm, was amplified using the primers BhCas12b-b-F and BhCas12b-b-R. The resulting two fragments were ligated using Gibson assembly, yielding pHT-BhCas12b. The synthesized sgRNA targeting *sacA*, which was expressed through P_{veg}, was amplified using the primers sacABhsg-F and sacABhsg-R, while the backbone of pHT-BhCas12b, was amplified using the primers sacABhsg-b-F and sacABhsg-b-R. The resulting two fragments were ligated using Gibson assembly, yielding the *sacA* deletion plasmid pHT-BhCas12b-AIO-sacA. To produce a plasmid for the deletion of *aprE* based on CRISPR-BhCas12b, we first used the pHT-BhCas12b-AIO-sacA plasmid as a template to carry out reverse PCR by using the primers pHT-aprEsg-F and pHT-aprEsg-R to obtain the sgRNA targeting *aprE*. Previously, we successfully constructed pB-aprEHA, which contained the 1000 bp homologous arms of *aprE*. Here, we amplified the homologous arms of *aprE* from pB-aprEHA using the primers pHT-aprEHA-F and pHT-aprEHA-R, while the backbone of the plasmid harboring BhCas12b and aprEsgRNA was amplified using the primers pHT-aprEHA-b-F and pHT-aprEHA-b-R. Then, the resulting two fragments were ligated using Gibson assembly, yielding pHT-BhCas12b-AIO-aprE. For CRISPR-AaCas12b-mediated *sacA* deletion in *B. subtilis*, bacterial codon-optimized AaCas12b was amplified using the primers AaCas12b-F and AaCas12b-R, while the backbone of pHT-BhCas12b-AIO-sacA, harboring the *sacA* homologous arm, was amplified using the primers AaCas12b-b-F and AaCas12b-b-R. Then, the resulting two fragments were ligated using Gibson assembly, yielding pHT-AaCas12b. Subsequently, the synthesized sgRNA targeting *sacA*, which was expressed through P_{veg}, was amplified using the primers sacAAasg-F and sacAAasg-R, while the backbone of pHT-AaCas12b, was amplified using the primers sacAAasg-b-F and sacAAasg-b-R. The resulting two fragments were ligated using Gibson assembly, yielding the *sacA*

deletion plasmid pHT-AaCas12b-AIO-sacA.

Plasmid construction for transcription repression in *B. subtilis* (CRISPRi):

Here, dBhCas12b was amplified using the primers pAX-dBhCas12b-F and pAX-dBhCas12b-R, while the integration plasmid backbone of pAX-dCas9 was amplified using primers pAX-dBhCas12b-b-F and pAX-dBhCas12b-b-R. The resulting two fragments were ligated using Gibson assembly, yielding the plasmid pAX-dBhCas12b. For the expression of BhsgRNA, BhsgRNA was amplified using the primers BhsgRNA-F and BhsgRNA-R, while the backbone of pDGT, harboring the P43-GFP expression cassette, was amplified using the primers BhsgRNA-b-F and BhsgRNA-b-R. The two fragments were ligated to replace P43-GFP with P_{veg}-BhsgRNA, resulting in pDGT-BhsgRNA. If targeting different positions of eGFP (such as G1), a pair of primers (such as G1-F and G1-R) can be designed for reverse PCR to replace the different N23 (pDGT-G1). The design of the sgRNA targeting promoters was the same as that for eGFP. The fluorescent-reporter plasmid pB-P43-eGFP was constructed in our previous studies. Fluorescent reporter plasmids with different promoters were constructed using the primers ylbP-F and ylbP-R, rpoB-F and rpoB-R, relA-F and relA-R, spoVG-F and spoVG-R, and sigW-F and sigW-R for reverse PCR, producing pB-PylbP-eGFP, pB-PrpoB-eGFP, pB-PrelA-eGFP, pB-PspoVG-eGFP, and pB-PsigW-eGFP, respectively.

Plasmid construction for diversifying expression in *B. subtilis*:

The lysine at the third position of eGFP was mutated to asparagine (K3N) using the primers K3N-F and K3N-R for reverse PCR to produce pB-P43-eGFP (K3N). The original (RBS+spacer, RS) on pB-P43-eGFP (K3N) was replaced with tailored RS (GGGGGGGGGGGGGG) using the primers mRS-F and mRS-R through reverse PCR, generating pB-P43-mRS-eGFP (K3N). An sgRNA targeting the tailored RS was amplified using the primers mRS-sg-F and mRS-sg-R, and the corresponding backbone pB-P43-mRS-eGFP (K3N) was amplified using the primers mRS-sg-b-F and mRS-sg-b-R, resulting in two fragments assembled to produce pB-P43-mRS-eGFPsg.

Plasmid construction for base editing in *B. subtilis* (dBhCas12b-based BEs):

Here, dBhCas12b was amplified using the primers pAX-cCDA-F and pAX-cCDA-R,

while the integration plasmid backbone of pAX-dCas9-CDA was amplified using primers pAX-cCDA-b-F and pAX-cCDA-b-R. The resulting two fragments were ligated using Gibson assembly, yielding the plasmid pAX-dBhCas12b-CDA. The CDA was amplified using the primers pAX-nCDA-F and pAX-nCDA-R, while the integration plasmid backbone of pAX-dBhCas12b was amplified using primers pAX-nCDA-b-F and pAX-nCDA-b-R. The resulting two fragments were ligated using Gibson assembly, yielding the plasmid pAX-CDA-dBhCas12b. The UGI was amplified using the primers pAX-UGI-F and pAX-UGI-R, while the integration plasmid backbone of the pAX-CDA-dBhCas12b was amplified using the primer pAX-UGI-b-F and pAX-UGI-b-R. The resulting two fragments were ligated using Gibson assembly, yielding the plasmid pAX-CDA-dBhCas12b-UGI. The other UGI was amplified using the primers pAX-2UGI-F and pAX-2UGI-R, while the integration plasmid backbone of pAX-CDA-dBhCas12b-UGI was amplified using the primers pAX-2UGI-b-F and pAX-2UGI-b-R. The resulting two fragments were ligated using Gibson assembly, yielding the plasmid pAX-CDA-dBhCas12b-UGI-UGI. For the expression of sgRNA, the sgRNA targeting *pksA*, which was expressed through P_{veg}, was amplified using primers pksABhsg-F and pksABhsg-R, while the backbone of pHYT was amplified using the primers pksABhsg-b-F and pksABhsg-b-R. The resulting two fragments were ligated using Gibson assembly, yielding pHY-pksA. The construction process for pHY-pksC was similar to that for pHY-pksA. For the construction of the dBhCas12b-based ABE, ABE8e was amplified using the primers pAX-8e-F and pAX-8e-R, while the integration plasmid backbone of pAX-dBhCas12b was amplified using the primers pAX-8e-b-F and pAX-8e-b-R. The resulting two fragments were ligated using Gibson assembly, yielding the plasmid pAX-ABE8e-dBhCas12b. pHY-sigE was constructed using pHY-pksA as the template for reverse PCR using the primers pHY-sigE-F and pHY-sigE-R to replace the sgRNA targeting *sigE*.

Plasmid construction for base editing in *E. coli* (dBhCas12b-based CBE):

For construction of the pKD-BhCBE-AIO plasmid, we performed the following operations with pKD46 as the template. First, the CDA-dBhCas12b-UGI fragment was amplified by the primers pKD-CDA-F and pKD-CDA-R using pAX-CDA-dBhCas12b-

UGI as template, and then the backbone was amplified by the primers pKD-CDA-b-F and pKD-CDA-b-R using pKD46 as template. Finally, the CDA-dBhCas12b-UGI fragment and backbone were stitched by Gibson assembly (homologous recombination enzymes α, β, and θ replaced by CDA-dBhCas12b-UGI), producing pKD-CDA-dBhCas12b-UGI. Then, the sgRNA expression cassette was amplified by the primers pKD-Bhsg-F and pKD-Bhsg-R using pHY-sigE (containing the sgRNA expression cassette targeting *sigE*) as a template. The backbone of sgRNA was amplified by the primers pKD-Bhsg-b-F and pKD-Bhsg-b-R using pKD-CDA-dBhCas12b-UGI as template. Finally, the two fragments were connected by Gibson assembly, yielding pKD-BhCBE-AIO. If targeting different sites (such as rpsE1), a pair of primers (such as rpsE1-F and rpsE1-R) could be used for reverse PCR to generate the new N23. The construction methods for other plasmids targeting different genes were similar. For the construction of the plasmid pBAD-ssTorA-sfGFP, a signal peptide targeting Tat-TorA was amplified by the primers ssTorA-F and ssTorA-R, and then the backbone was amplified by the primers ssTorA-b-F and ssTorA-b-R using pBAD-sfGFP as a template. Then, two fragments were ligated using Gibson assembly to produce the final plasmid pBAD-ssTorA-sfGFP. The bacterial codon-optimized BhCas12b and AaCas12b, as well as the corresponding sgRNAs (BhsgRNA and AasgRNA), were synthesized by GENEWIZ Inc., Ltd. (Wuxi, China).

Plasmid construction for base editing in *E. coli* (dSpCas9- and dFnCas12a-based CBEs):

For construction of the pKD-SpCBE-AIO plasmid, we performed the following operations with pKD-rpsE1 as the template. First, the dCas9 fragment was amplified by the primers pKD-dCas9-F and pKD-dCas9-R using pAX-dCas9 as template, and then the backbone was amplified by the primers pKD-dCas9-b-F and pKD-dCas9-b-R using pKD-rpsE1 as template. Finally, the dCas9 fragment and backbone were stitched by Gibson assembly, producing pKD-CDA-dCas9-UGI. Then, the sgRNA expression cassette was amplified by the primers pKD-Spsg-F and pKD-Spsg-R using pHT-lacA (containing the sgRNA expression cassette targeting *lacA*) as a template. The backbone of sgRNA was amplified by primers pKD-Spsg-b-F and pKD-Spsg-b-R using pKD-

CDA-dCas9-UGI as template. Finally, the two fragments were connected by Gibson assembly, yielding pKD-SpCBE-AIO. Taking *ptsG* targeting as an example, pKD-SpCBE-AIO was used as the template for reverse PCR using the primers SpptsG-F and SpptsG-R, generating pKD-SpCBE-ptsG. The construction method for other gene targeting plasmids was similar to that for pKD-SpCBE-ptsG.

For construction of the pKD-FnCBE-AIO plasmid, we performed the following operations with pKD-rpsE1 as the template. First, the FnCas12a fragment was amplified by the primers pKD-Cas12a-F and pKD-Cas12a-R using pHTsacA as template, and then the backbone was amplified by the primers pKD-Cas12a-b-F and pKD-Cas12a-b-R using pKD-rpsE1 as template. Finally, the FnCas12a fragment and backbone were stitched by Gibson assembly, producing pKD-CDA-FnCas12a-UGI. The plasmid pKD-CDA-FnCas12a-UGI was used as the template for reverse PCR using the primers E1006A-F and E1006A-R to produce pKD-CDA-dFnCas12a-UGI. Then, the crRNA expression cassette was amplified by the primers pKD-Fncr-F and pKD-Fncr-R using pHTsacA (containing the crRNA expression cassette targeting *sacA*) as a template. The backbone of crRNA was amplified by the primers pKD-Fncr-b-F and pKD-Fncr-b-R using pKD-CDA-dFnCas12a-UGI as template. Finally, the two fragments were connected by Gibson assembly, yielding pKD-FnCBE-AIO. Taking targeted *ptsG* as an example, pKD-FnCBE-AIO was used as the template for reverse PCR using the primers FnptsG-F and FnptsG-R, generating pKD-FnCBE-ptsG. The construction method for other gene targeting plasmids was similar to that for pKD-FnCBE-ptsG.

Supplementary Sequences

Key genetic parts (partial)

CDA-dBhCas12b-UGI-UGI

ATGACAGATGCCGAATACGTTCGCATCCACGAGAAGCTGGATATCTACACG

TTTAAAAAGCAGTTTTTAACAATAAGAAGAGCGTCTCCCACCGCTGCTAT

GTTCTTTTCGAACTGAAACGCAGAGGCGAAAGACGCGCTTGCTTCTGGGG

ATATGCCGTTAACAAACCGCAGTCCGGCACGGAACGCGGCATTCACGCCG

AGATCTTCAGCATCCGCAAGGTTGAGGAGTATCTTCGCGATAACCCGGGCC
AGTTTACGATCAACTGGTACAGCAGCTGGAGCCCGTGTGCCGATTGCGCCG
AAAAGATTCTTGAGTGGTACAACCAAGAACTTCGCGGCAATGGCCACACG
CTGAAAATCTGGGCTTGCAAGCTGTACTACGAGAAGAACGCCCGCAATCA
GATCGGACTTTGGAATCTTCGCGATAATGGCGTTGGACTTAACGTCATGGTC
TCCGAACACTACCAGTGCTGCCGCAAGATCTTCATCCAGTCCTCCCACAAC
CAGCTTAACGAAAACCGCTGGCTGGAGAAGACACTGAAACGCGCTGAAA
AGCGCCGCTCCGAGCTGAGCATCATGATCCAAGTTAAGATTCTTCATACGA
CGAAGAGCCCGGCCGTCGGTTCTGCAGCTTCTAGAATGGCTACGAGAAGC
TTCATTCTGAAGATCGAGCCGAACGAAGAAGTCAAAAAGGGACTTTGGAA
GACGCACGAAGTCCTTAATCATGGCATCGCTTATTATATGAACATTCTTAAG
CTGATCAGACAAGAGGCCATCTATGAGCACCATGAGCAAGATCCAAAGAA
CCCGAAGAAGGTTAGCAAGGCCGAGATTCAAGCCGAGCTGTGGGACTTCG
TTCTTAAAATGCAAAAGTGCAACTCCTTTACGCATGAAGTCGACAAGGATG
AAGTTTTTAACATCCTTCGCGAGCTTTATGAGGAACTGGTCCCAAGCTCCG
TCGAGAAAAAAGGAGAGGCCAACCAGCTGTCCAACAAGTTCCTTTATCCG
CTGGTCGATCCGAACTCCCAGTCCGGAAAAGGAACGGCCTCCAGCGGACG
CAAACCGCGCTGGTATAATCTTAAGATCGCTGGCGACCCGAGCTGGGAAGA
AGAGAAGAAGAAGTGGGAGGAAGACAAGAAAAAGGATCCGCTTGCCAAA
ATTCTTGGCAAGCTGGCCGAGTACGGACTTATCCCACTGTTCATCCCGTACA
CGGATAGCAACGAACCGATCGTCAAGGAGATCAAGTGGATGGAAAAAAGC
CGCAACCAAAGCGTCCGCAGACTTGACAAGGATATGTTCATCCAAGCTCTT
GAGAGATTTCTGAGCTGGGAGAGCTGGAATCTTAAAGTCAAGGAAGAGTA
CGAGAAAGTCGAGAAAGAATACAAAACACTTGAGGAACGCATCAAAGAA
GACATTCAAGCTCTTAAAGCTCTTGAACAGTATGAGAAGGAGCGCCAAGA
ACAACTGCTTCGCGACACACTTAACACAAACGAGTACCGCCTTAGCAAGC
GCGGACTTCGCGGATGGCGCGAGATCATCCAGAAGTGGCTTAAGATGGAC
GAGAACGAGCCGTCCGAGAAGTACCTTGAAGTTTTCAAAGATTATCAGAG
AAAACACCCGCGCGAAGCCGGCGATTATAGCGTTTACGAGTTCCTTAGCAA
GAAAGAAAACCACTTCATTTGGCGCAATCACCCGGAATACCCGTATCTTTAT

GCCACGTTTTGCGAGATTGACAAGAAGAAGGACGCTAAGCAGCAAGC
CACGTTTACGCTTGCTGACCCAATCAACCACCCGCTGTGGGTTAGATTTGA
AGAGCGCAGCGGCAGCAATCTGAATAAATATAGAATTCTTACAGAACAACT
TCATACGGAAAAACTTAAGAAGAAACTGACAGTCCAGCTGGATAGACTTAT
TTATCCAACAGAGAGCGGCGGCTGGGAGGAAAAGGGCAAGGTTGACATCG
TCCTTCTGCCGTCCCGCCAGTTCTACAACCAAATCTTTCTTGACATTGAGGA
GAAGGGCAAGCACGCCTTCACATACAAGGACGAATCCATCAAGTTCCCGC
TGAAGGGCACACTTGGCGGAGCTAGAGTTCAGTTCGACCGCGACCATCTT
CGCAGATATCCGCACAAGGTCGAAAGCGGCAATGTCGGCCGCATTTACTTC
AATATGACGGTCAACATCGAGCCGACGGAATCCCCGGTTAGCAAGTCTCTT
AAGATCCATCGCGACGATTTCCCGAAGGTCGTCAACTTCAAGCCAAAAGA
GCTGACAGAGTGGATTAAGGACAGCAAAGGAAAAAAGCTGAAGTCCGGC
ATCGAATCTCTTGAAATCGGCCTTCGCGTCATGAGCATCGCACTTGGACAA
AGACAAGCTGCTGCTGCCAGCATCTTTGAGGTTGTCGACCAGAAGCCGGA
TATCGAGGGCAAACTGTTCTTTCCAATTAAGGGCACAGAGCTTTACGCTGT
CCACCGCGCCTCCTTCAATATCAAACTGCCGGGAGAGACACTGGTTAAATC
CCGCGAAGTTCTTCGCAAGGCTAGAGAGGACAACCTTAAGCTGATGAATC
AGAAACTGAACTTCCTTAGAAATGTTCTTCACTTCCAACAGTTCGAGGATA
TCACGGAACGCGAGAAGAGAGTTACGAAGTGGATCAGCAGACAAGAAAA
CAGCGATGTCCCACTGGTCTACCAAGATGAGCTTATTCAGATTCGCGAACT
TATGTACAAGCCGTACAAAGACTGGGTCGCCTTTCTGAAACAGCTTCACAA
AAGACTTGAGGTCGAGATTGGCAAGGAAGTTAAGCACTGGCGCAAATCCC
TTAGCGATGGCCGCAAAGGCCTTTACGGAATCTCTCTTAAAAATATTGATGA
AATTGATCGCACACGCAAATTTCTGCTGCGCTGGTCTCTTAGACCGACAGA
ACCGGGCGAAGTCAGAAGACTTGAACCGGGCCAACGCTTTGCCATCGACC
AGCTGAACCATCTGAATGCTCTGAAGGAGGACCGCCTTAAGAAGATGGCT
AATACAATCATTATGCATGCCCTTGGATACTGCTATGACGTTAGAAAAAAGA
AGTGGCAAGCCAAGAATCCGGCTTGCCAAATCATTCTGTTTGCAGATCTTT
CCAACTACAATCCGTACGGCGAGAGAAGCCGCTTTGAGAATTCCAGACTTA
TGAAGTGGTCCCGCCGCGAGATTCCAAGACAAGTTGCTCTGCAAGGAGAG

ATTTACGGCCTTCAAGTTGGCGAAGTTGGAGCCCAGTTCAGCAGCAGATTC

CATGCTAAAACGGGAAGCCCGGGAATCAGATGCCGCGTTGTCACGAAGGA

GAAGCTTCAAGATAACCGCTTTTTCAAAAATCTGCAGCGCGAAGGCCGCCT

TACGCTTGACAAAATTGCCGTCCTTAAGGAGGGCGATCTGTACCCGGACAA

GGGCGGCGAGAAGTTCATCTCTCTTAGCAAGGACCGCAAATGCGTTACGA

CGCATGCCGCAATTAACGCCGCCCAGAATCTTCAAAAACGCTTCTGGACGC

GCACGCATGGCTTCTATAAGGTCTACTGTAAAGCCTACCAAGTTGACGGAC

AGACGGTCTATATCCCGGAAAGCAAGGATCAAAAGCAAAAGATCATCGAA

GAGTTCGGCGAGGGATACTTTATTCTTAAGGACGGCGTTTACGAATGGGTC

AACGCCGGAAAGCTGAAAATCAAGAAAGGCTCCTCCAAACAGTCCAGCTC

CGAACTGGTCGATAGCGATATTCTTAAGGACAGCTTTGATCTTGCCTCCGAG

CTGAAGGGCGAAAAGCTTATGCTGTATCGCGATCCGAGCGGCAATGTTTTT

CCAAGCGACAAGTGGATGGCCGCCGGCGTTTTCTTCGGAAAGCTGGAACG

CATTCTTATTAGCAAACTGACAAATCAGTACAGCATCTCCACGATCGAGGAT

GATTCCTCCAAGCAGTCCATGGGACCGAAGAAGAAGCGCAAGGTGGGGAC

CATGACGAATCTTAGCGACATCATCGAGAAGGAAACGGGCAAACAGCTTG

TCATCCAAGAAAGCATTCTTATGCTGCCGGAAGAGGTCGAAGAGGTCATCG

GCAACAAACCGGAAAGCGACATTCTTGTCCATACAGCCTACGATGAGAGC

ACGGATGAGAACGTCATGCTTCTGACGAGCGATGCCCCGGAATATAAACCG

TGGGCTCTTGTCATTCAAGATAGCAACGGCGAGAATAAGATCAAAATGCTT

TCAGGTTCTGAAACTCCTGGAACCAGTGAGTCTGCAACTCCAGAGTCTATG

ACGAATCTTAGCGACATCATCGAGAAGGAAACGGGCAAACAGCTTGTCAT

CCAAGAAAGCATTCTTATGCTGCCGGAAGAGGTCGAAGAGGTCATCGGCA

ACAAACCGGAAAGCGACATTCTTGTCCATACAGCCTACGATGAGAGCACG

GATGAGAACGTCATGCTTCTGACGAGCGATGCCCCGGAATATAAACCGTGG

GCTCTTGTCATTCAAGATAGCAACGGCGAGAATAAGATCAAAATGCTTTAA

Annotations:

CDA, dBhCas12b, UGI

sgRNA (targeting *sacA*)

GTTCTGTCTTTTGGTCAGGACAACCGTCTAGCTATAAGTGCTGCAGGGTGT

GAGAAACTCCTATTGCTGGACGATGTCTCTTACGAGGCATTAGCACCTGGC

TGATGCTCCCTCCATCCC

Annotations:

tracrRNA, crRNA, alternative 23 base target site

References

1 Xu, L., Liu, Y. & Han, R. BEAT: A python program to quantify base editing from sanger sequencing. *CRISPR J.* **2**, 223-229, (2019).
